# Explainable Machine Learning for Predicting Hospital Admission at Emergency Department Triage: Retrospective Cohort Study

**DOI:** 10.2196/98273

**Published:** 2026-11-10

**Authors:** Kalash Dave, Antony Robert, W Alton Russell, David L Buckeridge

**Affiliations:** 1 Department of Medicine, McGill University, 845 Sherbrooke Street West, Montreal, QC, H3A 0G4, Canada, 1 4384624410; 2 Department of Emergency Medicine, McGill University Health Centre, Montreal, QC, Canada; 3 School of Population and Global Health, McGill University, Montreal, QC, Canada

**Keywords:** emergency department crowding, triage, patient flow, machine learning, predictive analytics, explainable AI, clinical decision support systems, bed management, digital health, waiting time, length of stay

## Abstract

**Background:**

Emergency departments (EDs) are vital access points for urgent care, yet persistent overcrowding in health care systems worldwide compromises service quality and efficiency. Urban tertiary care centers are increasingly strained by surging patient numbers and scarce inpatient beds. Prior admission prediction studies mostly rely on limited triage features and discrimination metrics, neglecting operational factors, calibration, and explainability. Identifying likely admissions early could enable proactive resource allocation and shorter wait times.

**Objective:**

This study aimed to develop and evaluate machine learning models for predicting hospital admission at the end of triage using routinely collected structured data while addressing prior limitations regarding feature selection, model calibration, and explainability.

**Methods:**

We conducted a retrospective cohort study of adult ED visits between January 2015 and December 2022 at 2 academic hospitals in the McGill University Health Centre. We excluded visits with dispositions other than admission or discharge to ensure a binary outcome. Data were split into training (70%), validation (10%), and testing (20%) sets using stratified random sampling. We trained classifiers, including logistic regression (LR), decision trees, random forests, artificial neural networks, and gradient boosting algorithms (extreme gradient boosting [XGBoost], light gradient boosting machine, and categorical boosting), using demographics, vital signs, chief complaints, comorbidities, and historical hospitalization frequency available at triage. Sensitivity analyses tested exclusion criteria and temporal data shifts, while subgroup analyses examined differences by age, sex, and site. Performance was evaluated using area under the receiver operating characteristic curve (AUC-ROC), sensitivity, specificity, and calibration. We also performed a decision threshold analysis to assess operational flexibility and used Shapley additive explanations (SHAP) values to interpret feature contributions.

**Results:**

The final cohort included 526,145 visits (mean age 50.9, SD 21.1 years; 272,767/526,145, 51.8% female), with 91,363 patients (17.4%) admitted. The XGBoost model achieved the highest performance, with an AUC-ROC of 0.86 (95% CI 0.858‐0.864), a sensitivity of 0.67, and a specificity of 0.86, outperforming the LR baseline (AUC 0.84). Key predictors identified by SHAP values included age, Canadian Triage and Acuity Scale scores, arrival mode, and historical hospitalization frequency. Subgroup analyses confirmed strong performance across age, sex, and sites. Sensitivity analyses evaluating alternative outcome dispositions and chronological data splits confirmed overall model stability. Threshold analysis showed that adjusting the decision threshold to the 0.70 to 0.75 range improved the positive predictive value and did not disproportionately reclassify older or higher-acuity patients.

**Conclusions:**

In urban tertiary care settings, structured triage data available at triage can effectively anticipate hospital admissions. By incorporating broad feature sets and operational variables, models such as XGBoost offer a feasible, explainable tool for early decision-making. Such tools could improve coordination between emergency and inpatient services and ease overcrowding. Future work should prioritize prospective validation and real-time clinical workflow integration.

## Introduction

Emergency departments (EDs) are the foundation of acute care delivery in modern health care systems, providing essential services for patients requiring immediate medical attention [1]. Originally designed for emergency stabilization, contemporary EDs have evolved into multifaceted clinical units that manage diverse patient needs while serving as critical safety nets for vulnerable populations [2,3]. This transformation has occurred alongside steadily increasing patient volumes globally, creating significant strain on existing infrastructure and resources [4]. The resulting overcrowding manifests most acutely in urban hospitals, where operational capacity thresholds are routinely exceeded [5]. Within Canada’s health care network, for example, these pressures have reached critical levels. Admitted patients experience prolonged waits for inpatient placement that substantially exceed internationally recommended benchmarks [6].

The patient care pathway through EDs follows a defined sequence, beginning with standardized triage assessment using the Canadian Triage and Acuity Scale (CTAS) [7] and progressing through physician evaluation, diagnostic testing, and ultimate disposition determination. Each transitional phase presents opportunities for delay accumulation, creating bottlenecks that compromise timely care delivery [8]. Patients frequently experience extended waits for initial physician assessment following triage, while those requiring hospital admission often remain in ED treatment spaces for prolonged periods awaiting inpatient bed assignment [9]. These operational challenges transcend simple efficiency metrics, representing fundamental breakdowns in a system designed to deliver prompt, life-saving interventions [10].

For patients, these systemic delays correlate with measurable declines in care quality and clinical outcomes [11]. Emergency care providers face parallel challenges, with documented increases in workforce stress and clinical decision-making difficulties under crowded working conditions [12]. These interrelated consequences emerge from three primary sources: growing patient care demands, operational inefficiencies in care delivery processes, and limited inpatient bed availability to accommodate admitted patients [13]. The cumulative impact creates substantial financial burdens for health care systems while compromising patient safety and care quality [1,10,14].

Existing strategies to mitigate ED overcrowding have demonstrated only partial success [15]. Internationally, front-end solutions prioritize organizing or diverting nonemergent patients before they arrive, using interventions such as ambulance diversion to alternative care pathways [16] and front-door streaming of low-acuity cases to adjacent general practices [17]. Despite these external routing efforts, tertiary care centers continue to face massive volumes of undifferentiated patients who require formal ED evaluation. Therefore, there remains a critical need for internal, data-driven approaches that can anticipate inpatient admission requirements early in the ED visit cycle. Most existing predictive models rely on a narrow set of triage-level features and focus primarily on discrimination performance, with limited attention to calibration, other relevant performance metrics, interpretability, or subgroup performance.

To address these gaps, this study asked the following research question: Can machine learning (ML) models, using only structured data available at triage, accurately predict hospital admissions and provide explainable insights for early decision-making? We addressed this question in the context of 2 large tertiary care EDs in Canada. We hypothesized that incorporating a comprehensive set of clinical and operational data would produce highly accurate and well-calibrated predictions. Furthermore, we aimed to apply explainable AI methods to clarify the underlying factors driving these predictions, making the tool practical for real-world clinical use.

## Methods

### Study Design, Data Sources, and Population

This retrospective cohort study analyzed electronic health records (EHRs) from 2 academic tertiary care EDs within the MUHC network: the Montreal General Hospital (MGH) and the Royal Victoria Hospital (RVH). These 2 cases in Canada were specifically chosen because they capture a diverse urban patient population and case mix while operating under a single health care network, which ensures high consistency in clinical practices and data recording. This study is reported in accordance with the TRIPOD (Transparent Reporting of a Multivariable Prediction Model for Individual Prognosis Or Diagnosis)+AI guidelines [18]. The completed checklists for the abstract and the main paper are available in Multimedia Appendix 1 and Multimedia Appendix 2, respectively. Data were extracted from the institutional clinical data warehouse, which aggregates information from multiple hospital systems using standardized protocols.

The study encompassed all consecutive ED visits between January 1, 2015, and December 31, 2022 (N=1,359,344), representing a complete 8-year period of adult (more than 18 years of age) patient encounters; no a priori sample size calculation was performed as we used the entire available population meeting inclusion criteria. Pediatric patients, visits to other network hospitals, and invalid records were excluded to ensure consistency in care delivery patterns and data capture methods.

The primary outcome was hospital admission status, derived from electronic disposition records. After excluding 620 invalid records, initial analysis of the remaining 629,247 visits revealed the following distribution: discharged (434,782/629,247, 69.1%), admitted (91,363/629,247, 14.52%), left without being seen (LWBS; 53,194/629,247, 8.45%), transferred (15,574/629,247, 2.48%), deceased (1,203/629,247, 0.19%), and other dispositions (33,131/629,247, 5.26%). For binary classification modeling, we created a dichotomous outcome where discharged cases were coded as 0 (434,782/526,145, 82.6% of the final dataset) and admitted cases as 1 (91,363/526,145, 17.4% of the final dataset). To ensure methodological validity in predicting admission likelihood, we excluded cases with disposition pathways fundamentally different from our primary objective. LWBS patients were excluded because their failure to complete the physician assessment made potential admission status indeterminate, which could introduce outcome misclassification bias. Transferred patients were excluded due to the inability to ascertain their final disposition at receiving facilities, potentially compromising outcome variable integrity. Other outcomes were excluded (deceased and other dispositions) to maintain a binary classification framework. To assess the potential impact of these exclusions on model performance, we conducted a sensitivity analysis using alternative outcome definitions; see the Sensitivity Analysis section for details. Figure 1 illustrates the step-wise inclusion and exclusion criteria applied to ED visits at the MUHC, resulting in a final sample size of N=526,145 visits.

**Figure 1.**
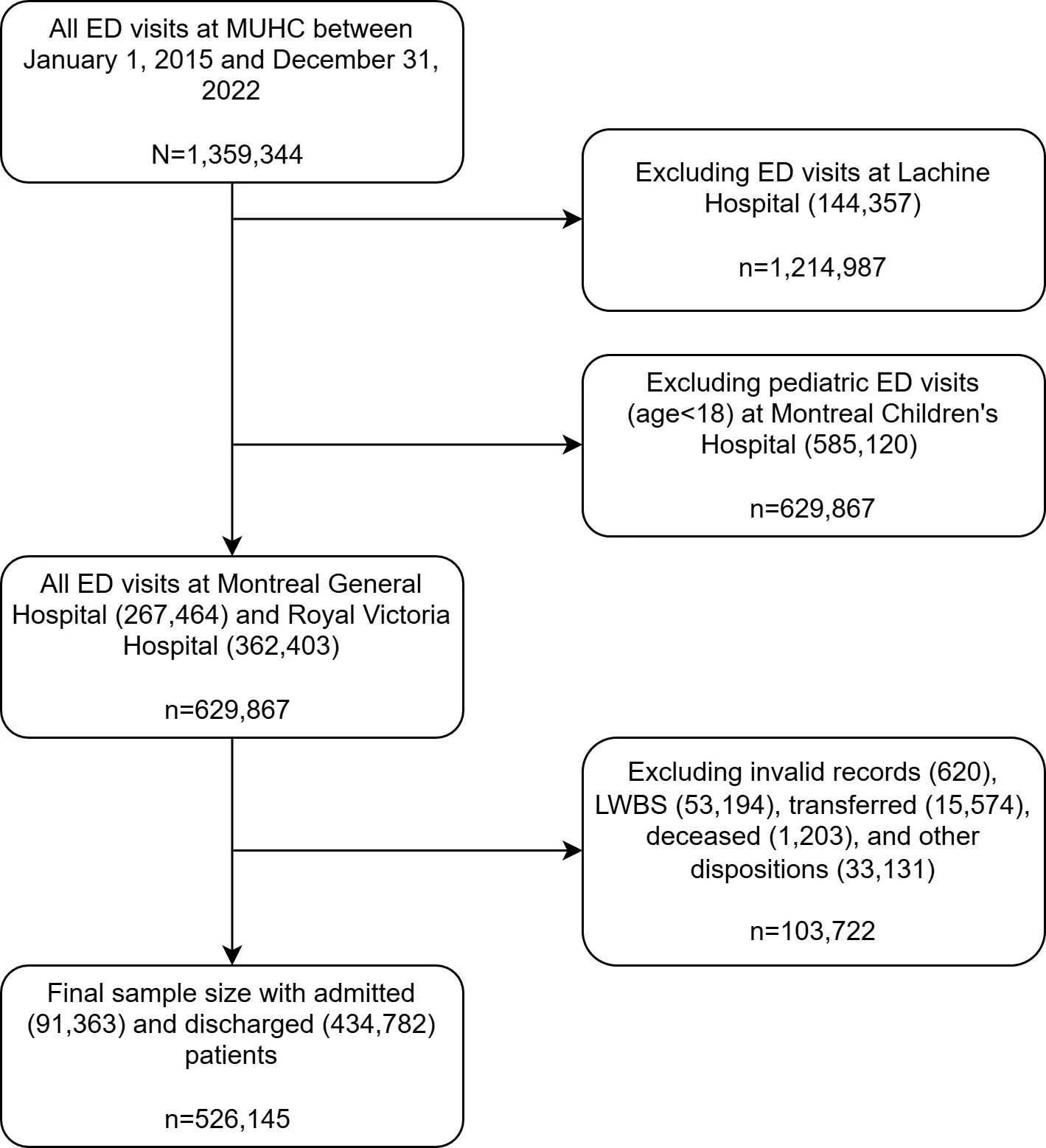
Flow diagram showing inclusion and exclusion criteria for emergency department visits in the study. ED: emergency department; LWBS: left without being seen; MUHC: McGill University Health Centre.

### Ethical Considerations

The research protocol (Protocol Number: 2024‐10385) received ethical approval from the Research Ethics Board (REB) of the McGill University Health Centre (MUHC). As this was a retrospective study using deidentified administrative data, the requirement for individual patient informed consent was waived by the REB. There was no patient or public involvement in the design, conduct, reporting, or dissemination of this research.

### Data Preparation

Initial data cleaning involved removing columns that contained no meaningful clinical information or administrative entries. We transformed the disposition outcome into a binary variable, coding admitted patients as 1 and discharged patients as 0, while excluding other disposition categories as detailed in the Study Design, Data Sources, and Population section. Twenty-one preexisting comorbidities were incorporated as binary features derived from patients’ medical histories. Using stratified random sampling, we partitioned the dataset into training (70%), validation (10%), and test sets (20%), at the encounter level, preserving the original outcome distribution (17.4% admissions vs 82.6% discharges) across all subsets. This approach maintained the natural class imbalance while ensuring each subset remained representative of the population distribution. A strict chronological split (Training: 2015 to 2020, Validation: 2021, and Testing: 2022) was also conducted as a sensitivity analysis to assess the impact of temporal data leakage; see the Sensitivity Analysis section for details. All performance metrics were calculated on the held-out test set, with AUC-ROC CIs derived using the analytical computation method [19,20].

The preprocessing function handled both feature engineering and data transformations. Temporal features such as arrival hour and day of the week were derived from the patient’s arrival timestamp data. The 159 unique triage chief complaints (TCCs) were categorized into medically relevant groups (eg, cardiovascular and respiratory) based on organ systems and clinical specialties (Table S7 in Multimedia Appendix 3). The vital sign measurements demonstrated Missing Not At Random (MNAR) patterns [21], where missingness was intrinsically linked to observed clinical factors, such as the triage nurse’s assessment of patient alertness or symptom presentation. For example, Glasgow Coma Scale (GCS) tests [22] were routinely omitted for alert patients without symptoms of cognitive impairment. Such informative missingness reflects a fundamental characteristic of ED workflows, as the absence of specific measurements may itself indicate clinical assessments of patient stability. To capture this information, we created a “Missing vital signs tests” feature that quantified the number of missing tests per patient. To maintain modeling integrity, we applied the preprocessing function independently to the training, validation, and test sets. This strict separation prevented data leakage [23] by ensuring no information from any set influenced transformations in another set.

To assess the model’s operational viability, we further analyzed the validation set to evaluate how changing the classification decision boundary affects patient selection. We identified patients whose predicted status changed from “admit” to “not admit” as the threshold increased from the default 0.5. We then analyzed the demographic (Age) and clinical (CTAS) characteristics of these reclassified patients to determine if higher precision thresholds inadvertently introduced bias against specific vulnerable subgroups.

### Feature Selection and Description

The predictive model incorporated clinical and administrative features available after initial triage. Three key hemodynamic parameters were computationally derived from blood pressure (BP) measurements. Mean arterial pressure (MAP), a critical indicator of end-organ perfusion, was calculated using MAP=(SBP+2×DBP)/3 [24], where SBP and DBP represent systolic and diastolic BP, respectively. Pulse pressure (PP), an established marker of arterial stiffness, was determined as the arithmetic difference between systolic and diastolic pressures (PP=SBP−DBP) [24]. The BP ratio, a less conventional but physiologically meaningful metric [25], was computed as the quotient of systolic to diastolic pressure (SBP /DBP).

The TCC, which was originally documented with approximately 159 unique entries, was categorized into 15 clinically meaningful groups. For instance, cardiovascular complaints included chest pain (anginal and non-anginal), palpitations, syncope, and cardiac arrest, while neurological complaints included seizure, tremors, and ataxia. This categorization preserved critical clinical information while reducing dimensionality, with final categories spanning cardiovascular, respiratory, gastrointestinal, neurological, musculoskeletal, trauma, genitourinary, ENT and ophthalmological, dermatological, psychiatric, infectious, metabolic and endocrine, toxicological, general or systemic, and procedural or follow-up. Comorbidity burden was assessed through 21 binary variables adapted from the Charlson Comorbidity Index [26].

Feature selection was guided by 3 criteria: (1) availability at initial triage to enable early decision-making, (2) clinical relevance based on prior emergency medicine literature linking the features to admission likelihood, and (3) inclusion of physiological, epidemiological, and systems-level factors known to affect patient disposition. Table 1 lists the predictor variables with their types and descriptions.

**Table 1.** List of input features used in predictive modeling.

Feature	Type	Description
Demographics		
Sex	Categorical	Biological sex (female, male, and unknown)
Age	Numeric	Patient’s age in years
Arrival mode^a^	Categorical	Mode of arrival: walk-in, ambulance, etc
Patient origin^a^	Categorical	Origin before ED^b^ visit: home, hospital, clinic, workplace, etc
Site	Categorical	Hospital site: MGH^c^ or RVH^d^
Triage		
CTAS^e^	Ordinal	CTAS (1: resuscitation, 2: emergent, 3: urgent, 4: less urgent, 5: nonurgent)
Triage chief complaint^f^	Categorical	Chief complaint category (eg, cardiovascular and dermatological)
Oxygen saturation	Numeric	Peripheral capillary oxygen saturation (SpO_2_%)
Temperature	Numeric	Body temperature (°C)
Respiration rate	Numeric	Breaths per minute
Pulse rate	Numeric	Heart rate (beats per minute)
DBP^g^	Numeric	BP^h^ (mmHg)
SBP^i^	Numeric	BP (mmHg)
Glasgow Coma Scale	Numeric	Consciousness assessment (3‐15 scale)
Glucose level	Numeric	Blood glucose (mmol/L)
Pain score	Numeric	Pain intensity (0: none-10: worst possible)
MAP^j^	Numeric	Derived from BP
PP^k^	Numeric	Derived from BP
BP ratio	Numeric	Derived from BP
Missing vital signs count	Ordinal	Number of triage vital sign tests not done
Temporal		
Day of month	Numeric	Day of arrival (1-31)
Month of arrival	Numeric	Month of arrival (1-12)
Hour of arrival	Numeric	Hour of arrival (0‐23)
Day of week	Numeric	Day of arrival (0: Monday-6: Sunday)
Is weekend	Binary	Arrival on Saturday or Sunday (yes or no)
Season of arrival	Categorical	Season (autumn, winter, spring, and summer) derived from month
Patient history		
ED visits past 12 months	Numeric	Number of ED visits in the past 1 year
Short stays hospital	Numeric	Prior inpatient hospitalizations in the past 5 years
Day surgeries hospital	Numeric	Outpatient surgeries in the past 5 years
System factor		
Arrivals past 60 minutes	Numeric	Number of ED arrivals at the site in the past 1 hour
Comorbidities		
Comorbidity present	Categorical	Presence of at least 1 comorbidity (yes or no)
Total comorbidities	Ordinal	Count of comorbidities per patient
Hypertension	Binary	Presence (yes or no)
Arrhythmias	Binary	Presence (yes or no)
Renal disease	Binary	Presence (yes or no)
Mild liver disease	Binary	Presence (yes or no)
Moderate or severe liver disease	Binary	Presence (yes or no)
Diabetes mellitus without complications	Binary	Presence (yes or no)
Diabetes mellitus with complications	Binary	Presence (yes or no)
Chronic pulmonary disease	Binary	Presence (yes or no)
Congestive heart failure	Binary	Presence (yes or no)
Peripheral vascular disease	Binary	Presence (yes or no)
Deficiency anemias	Binary	Presence (yes or no)
Fluid and electrolytes abnormality	Binary	Presence (yes or no)
Myocardial infarction	Binary	Presence (yes or no)
Cerebrovascular disease	Binary	Presence (yes or no)
Dementia	Binary	Presence (yes or no)
Connective tissue disease–rheumatic disease	Binary	Presence (yes or no)
Peptic ulcer disease	Binary	Presence (yes or no)
Paraplegia and hemiplegia	Binary	Presence (yes or no)
Cancer	Binary	Presence (yes or no)
Metastatic carcinoma	Binary	Presence (yes or no)
AIDS and HIV	Binary	Presence (yes or no)

a See Table S8 in Multimedia Appendix 3 for the list of all unique values for this feature.

b ED: emergency department.

c MGH: Montreal General Hospital.

d RVH: Royal Victoria Hospital.

e CTAS: Canadian Triage and Acuity Scale.

f See Table S7 in Multimedia Appendix 3 for detailed categorization.

g DBP: diastolic blood pressure.

h BP: blood pressure.

i SBP: systolic blood pressure.

j MAP: mean arterial pressure.

k PP: pulse pressure.

### ML Models

We developed supervised binary classification models to predict hospital admission from the ED, with all models generating both class labels and probability estimates. Several algorithms were implemented and compared: logistic regression (LR) served as an interpretable baseline, while decision trees (DTs) and random forests (RFs) provided nonlinear alternatives. Gradient boosting approaches included extreme gradient boosting (XGBoost), light gradient boosting machine (LGBM), and categorical boosting (CatBoost), each offering distinct advantages in handling various data types. To capture complex nonlinear relationships, we implemented 2 artificial neural network (ANN) architectures with multilayer perceptron (MLP) configurations. The first had 2 hidden layers with 100 neurons in the first layer and 50 in the second (MLP [100, 50]). The second had 3 hidden layers with 100 neurons in the first layer, 50 in the second, and 25 in the third (MLP [100, 50, 25]).

The dataset was partitioned into training (70%), validation (10%), and test (20%) subsets using stratified random sampling to maintain consistent outcome distributions across all splits (Figure 2). The training set was used for model development and hyperparameter tuning, while the validation set was used to assess overfitting or underfitting before final testing. After selecting the best model, we retrained it on the full training set and evaluated it on the validation set. Although retraining on both training and validation sets before testing is a valid approach, we maintained a strict separation to preserve the integrity of the final evaluation, as the 10% validation set was sufficient for intermediate assessment.

**Figure 2.**
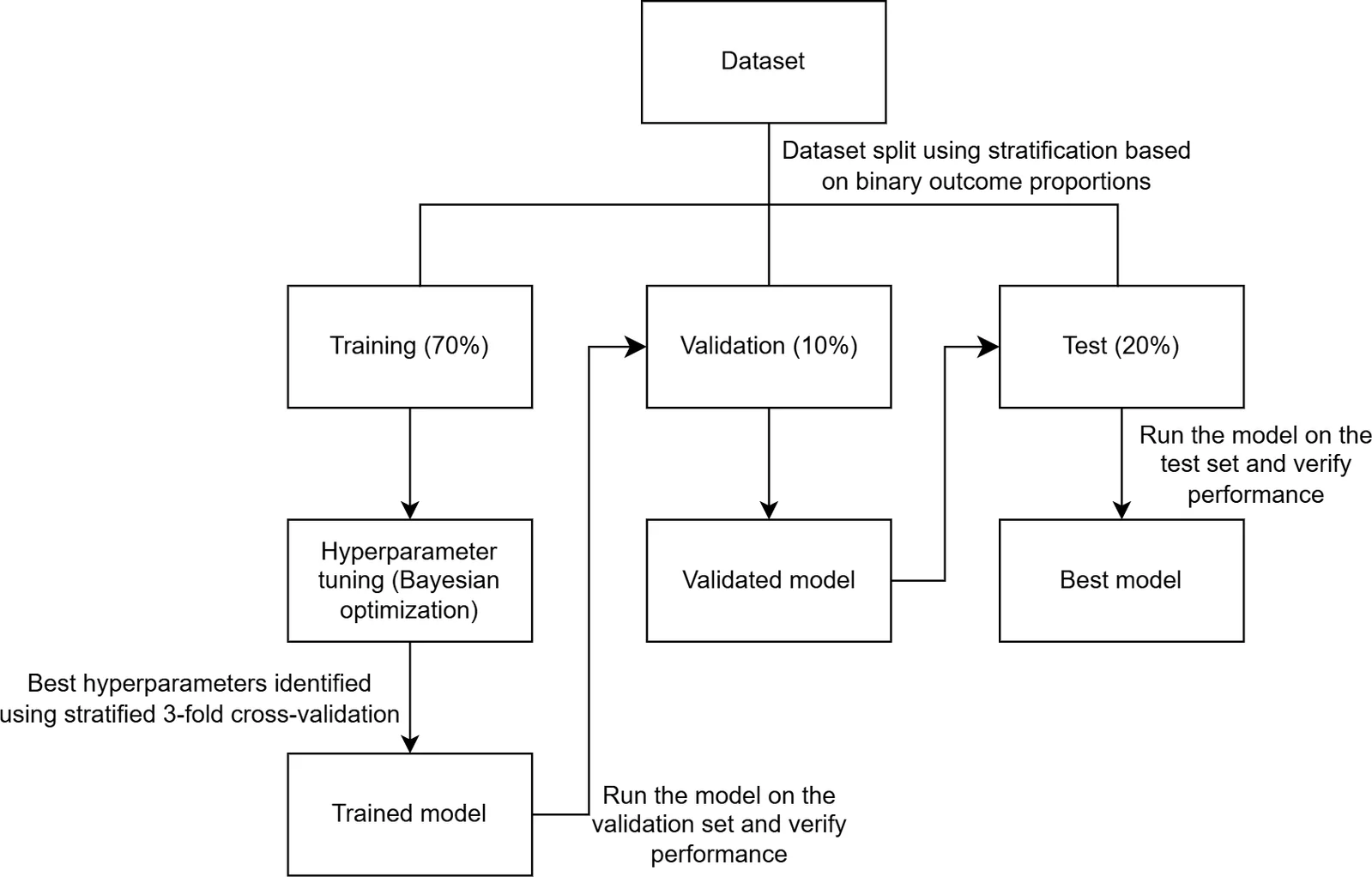
Machine learning workflow for model training, validation, and testing.

All feature transformations, including imputation and encoding, were systematically implemented using scikit-learn’s ColumnTransformer, which was integrated with each classifier within a unified sklearn Pipeline [27]. This methodology guaranteed that preprocessing steps remained properly encapsulated with the model while enabling complete workflow serialization. Cyclical transformations of temporal variables using sine and cosine encoding to capture periodicity were tested but did not improve performance, so they were not retained. Similarly, scaling numeric variables with RobustScaler, StandardScaler, QuantileTransformer, or PowerTransformer showed no meaningful gains and was excluded to preserve interpretability. Preprocessing varied by algorithm and feature type. Tree-based models (DT, RF, LGBM, CatBoost, and XGBoost) processed raw missing numerical and binary values directly without imputation. This leveraged native sparsity-aware splitting [28] to preserve the MNAR pattern. Categorical features were encoded directly. For models requiring complete data (LR and MLP), missing numerical variables were imputed using the median. Missing binary, ordinal, and categorical values were imputed using the most frequent training value or a dedicated “unknown” category.

We addressed class imbalance through algorithm-specific techniques: LR, DT, and RF used class weighting (*balanced* or *balanced*_*subsample* parameters), while XGBoost and LGBM used instance weighting via the *scale_pos_weight* parameter [28,29]. CatBoost implemented automatic class balancing via the *auto_class_weights* [30] parameter. ANNs were implemented using the MLPClassifier from the scikit-learn library. As this implementation does not support the *class_weight* parameter, class imbalance was addressed through resampling techniques. Consistent with prior findings that hybrid resampling strategies outperform individual approaches of either oversampling the minority or undersampling the majority [31], Synthetic Minority Oversampling Technique (SMOTE)–Tomek was used to balance class distributions in the training data. SMOTE-Tomek integrates SMOTE [31], which synthetically generates minority class samples, with Tomek links [32], which remove borderline samples from opposing classes that are each other’s nearest neighbors, thereby reducing class overlap and enhancing decision boundary clarity [33].

Following hyperparameter tuning, models were retrained on the full training set using optimized parameters and evaluated on the validation set (Figure 2). The best-performing model underwent final assessment on the held-out test set using comprehensive metrics including discrimination measures (AUC-ROC and area under the precision-recall curve [AUC-PR]), classification performance (accuracy, balanced accuracy, sensitivity, specificity, positive predicted value [PPV], negative predicted value [NPV], utility-weighted scores: *F*_0.5_-score, *F*_1_-score, and *F*_2_-score), calibration statistics (log loss and Brier score), and agreement measures (Matthews correlation coefficient and Cohen’s kappa) [27]. To enhance interpretability, we conducted feature importance analysis using Shapley additive explanations (SHAP) values, visualized through beeswarm plots that illustrate feature contributions to model outputs [34,35].

### Hyperparameter Tuning

Hyperparameter optimization was conducted using the Bayesian search method from the scikit-optimize library [36]. Tuning was performed via stratified 3-fold cross-validation, with the objective function designed to maximize the AUC-ROC score. An early stopping mechanism was implemented to monitor incremental improvements in AUC-ROC, terminating the search if no improvement greater than 0.001 was observed over 20 consecutive iterations. This conservative threshold ensured continued optimization only when clinically meaningful gains in discriminative performance were possible. The process continued until either the maximum number of iterations (n_iter =50) was reached, or the early stopping criterion was met.

In practice, the optimal set of hyperparameters was typically identified within the first 20 iterations across all models, as illustrated by the convergence plots in Figure S1 in Multimedia Appendix 3. The complete list of hyperparameters tuned is provided in Table S5 in Multimedia Appendix 3.

### Software and Libraries

The programming language Python (version 3.11.7; Python Software Foundation) was used throughout this study for model development, with implementations created using Jupyter Notebook (version 7.3.3; Project Jupyter) within VS Code (version 1.84.2; Microsoft Corp). Database connectivity was established via SQLAlchemy (version 2.0.36; Michael Bayer) to interface with SQL Server Management Studio (version 18.9.2; Microsoft Corp), enabling efficient data extraction into pandas (NumFOCUS; version 2.2.3) dataframes for subsequent processing and analysis.

The ML implementation leveraged several key libraries, including scikit-learn (version 1.5.2) for LR, RF, and ANNs; SHAP (version 0.46.0) for model interpretation; matplotlib (version 3.9.2; John D. Hunter) and seaborn (version 0.13.2; Michael Waskom) for visualizations; and scikit-optimize (version 0.10.2) for hyperparameter tuning and confidenceinterval (version 1.0.5) for CIs of AUC-ROC scores. Gradient boosting implementations used XGBoost (version 2.1.3), LGBM (version 4.5.0), and CatBoost (version 1.2.7).

## Results

### Characteristics of the Study Population

The study cohort comprised 526,145 unique adult ED visits, representing 268,496 unique patients, across 2 tertiary care centers between January 2015 and December 2022. Using stratified random sampling, the dataset was partitioned into training (n=368,301), validation (n=52,088), and test (n=105,756) sets. Baseline characteristics demonstrated good balance between splits, with less than 1% absolute difference in key features across sets, confirming the effectiveness of the stratification approach. The data distribution of demographic, triage, temporal, and comorbidity features across the study population is detailed in Table S1, Table S2, Table S3, and Table S4 in Multimedia Appendix 3. We prioritized clinically and prognostically significant categories for high-cardinality features (eg, arrival mode, patient origin, and TCC).

### Model Performance Comparison

Among all evaluated models, XGBoost was selected as the primary model due to its superior overall discrimination on the test set, achieving the highest AUC-ROC (0.8608, 95% CI 0.8580‐0.8635), AUC-PR (0.5863), and *F*_1_-score (0.5717). The complete receiver operating characteristic and precision-recall curves comparing the top-performing models are provided in Figure S3 and Figure S4 in Multimedia Appendix 3. While the model demonstrated high discriminative ability, calibration curves (Figure S5 in Multimedia Appendix 3) indicated a tendency to overestimate admission probabilities at higher risk thresholds. Although RF and CatBoost showed comparable AUC-ROC values (0.8509 and 0.8594, respectively), their performance was slightly lower in precision, *F*_1_-score, and log loss. Notably, the MLP-ST (multilayer perceptron with SMOTE-Tomek) models exhibited competitive results in terms of specificity and precision, particularly the deeper architecture (MLP-ST [100, 50, 25]), which achieved the highest specificity (0.9110) and precision (0.5435) among all models. However, both MLP-ST models underperformed in recall (0.5805 and 0.5045) and AUC-PR, suggesting a trade-off where the model favors correct identification of the negative class at the expense of missing more true positives. In contrast, LGBM achieved the highest recall (0.8224), but this came with reduced precision (0.3908), highlighting the classic precision-recall trade-off. While LR and DT models served as useful baselines, their overall predictive power lagged behind ensemble methods.

The performance metrics are presented in Table 2, with additional metrics including balanced accuracy, NPV, F-Beta (F_0.5_ and *F*_2_)-scores, and Cohen’s kappa, provided in Table S6 in Multimedia Appendix 3. All threshold-dependent metrics were calculated using a default threshold of 0.5 for consistency. Alternative thresholds could be considered but involve trade-offs (eg, increasing precision at the expense of recall) and may be explored further based on clinical or operational priorities.

**Table 2.** Performance metrics for all ML^a^ models.

Metrics	LR^b^	DT^c^	RF^d^	XGBoost^e^	LGBM^f^	CatBoost^g^	MLP-ST^h^ (100, 50)	MLP-ST (100, 50, 25)
AUC-ROC^i^ (95% CI)	0.8385 (0.8355-0.8416)	0.8264 (0.8233-0.8295)	0.8509 (0.8480-0.8537)	0.8608 (0.8580-0.8635)	0.8569 (0.8541-0.8597)	0.8594 (0.8566-0.8622)	0.8361 (0.8331-0.8390)	0.8423 (0.8393-0.8452)
AUC-PR^j^	0.5274	0.5021	0.5610	0.5863	0.5768	0.5795	0.5131	0.5297
Accuracy	0.7659	0.7330	0.8226	0.8259	0.7466	0.7725	0.8191	0.8404
Sensitivity (recall)	0.7522	0.7720	0.6378	0.6691	0.8224	0.7923	0.5805	0.5045
Specificity	0.7688	0.7248	0.8614	0.8589	0.7306	0.7684	0.8692	0.911
PPV^k^ (precision)	0.4060	0.3709	0.4917	0.4991	0.3908	0.4182	0.4825	0.5435
*F*_1_-score	0.5274	0.5010	0.5553	0.5717	0.5298	0.5475	0.5270	0.5233
Log loss	0.5047	0.5121	0.3971	0.3792	0.5040	0.4690	0.3857	0.3593
Brier score	0.1640	0.1729	0.1257	0.1208	0.1684	0.1544	0.1240	0.1133

a ML: machine learning.

b LR: logistic regression.

c DT: decision tree.

d RF: random forest.

e XGBoost: extreme gradient boosting.

f LGBM: light gradient boosting machine.

g CatBoost: categorical boosting.

h MLP-ST: multilayer perceptron with SMOTE-Tomek.

i AUC-ROC: area under the receiver operating characteristic curve.

j AUC-PR: area under the precision-recall curve.

k PPV: positive predictive value.

### Interpretation of Key Predictors

Figure 3 illustrates the contribution of the 8 most predictive features in the XGBoost model predicting hospital admission from ED visits (n=105,756). Features are ordered from top to bottom by their average impact (mean absolute SHAP value).

**Figure 3.**
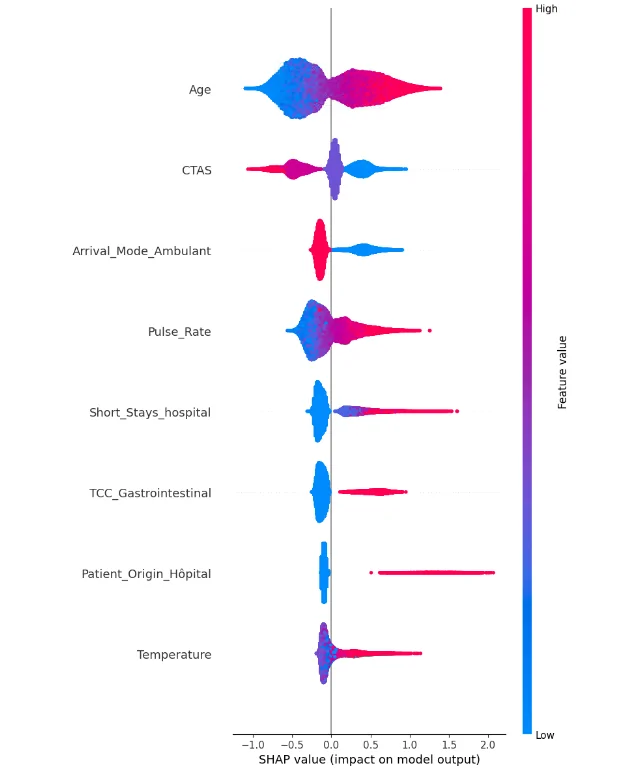
Shapley additive explanations (SHAP) beeswarm plot showing the top 8 predictors. CTAS: Canadian Triage and Acuity Scale; TCC: triage chief complaint.

Age emerged as the most influential feature, with a clear trend of increasing admission likelihood among older patients. This finding highlights the role of age as an independent predictor, rather than merely a surrogate for clinical complexity. The CTAS demonstrated strong predictive power, as lower scores (indicating higher severity) were strongly linked to admission, reinforcing its utility in identifying high-acuity patients. Arrival mode further differentiated risk, with ambulant patients (those arriving by themselves) having lower admission probabilities than those arriving by ambulance, likely reflecting differences in illness severity. Among vital sign measures, elevated pulse rates and higher temperatures were both independently associated with increased admission likelihood. The analysis also revealed 3 additional clinically significant predictors: Patients with more frequent hospitalizations in the past 5 years (Short_Stays_hospital) showed elevated admission rates, highlighting prior health care usage as a marker of medical vulnerability; gastrointestinal complaints at triage (TCC_Gastrointestinal) including abdominal pain, nausea, vomiting, or gastrointestinal bleeding, predicted higher admissions due to their association with conditions requiring diagnostic evaluation or intervention; and interhospital transfers (Patient_Origin_Hôpital) demonstrated significantly higher admission rates, emphasizing this predictor’s role as an indicator of case complexity exceeding referring facility capabilities. A comprehensive visualization detailing the global feature importance and directional impact of all variables included in the model is available in Figure S2 in Multimedia Appendix 3.

The SHAP waterfall plot (Figure 4) illustrates how individual features contribute to a specific patient visit’s prediction, showing how each feature shifts the output away from the model’s baseline, or base value [34]. To protect patient privacy, feature values shown in the individual explanation plots represent a hypothetical patient profile. In this case, the base value *E*[*f*(*x*)] is *−*0.958, representing the average model output across the dataset. The final prediction for this patient, denoted by *f*(*x*), is 1.152, indicating a likely admission. Features with positive SHAP values (shown in red) push the prediction toward admission, while those with negative values (in blue) pull it toward discharge.

**Figure 4.**
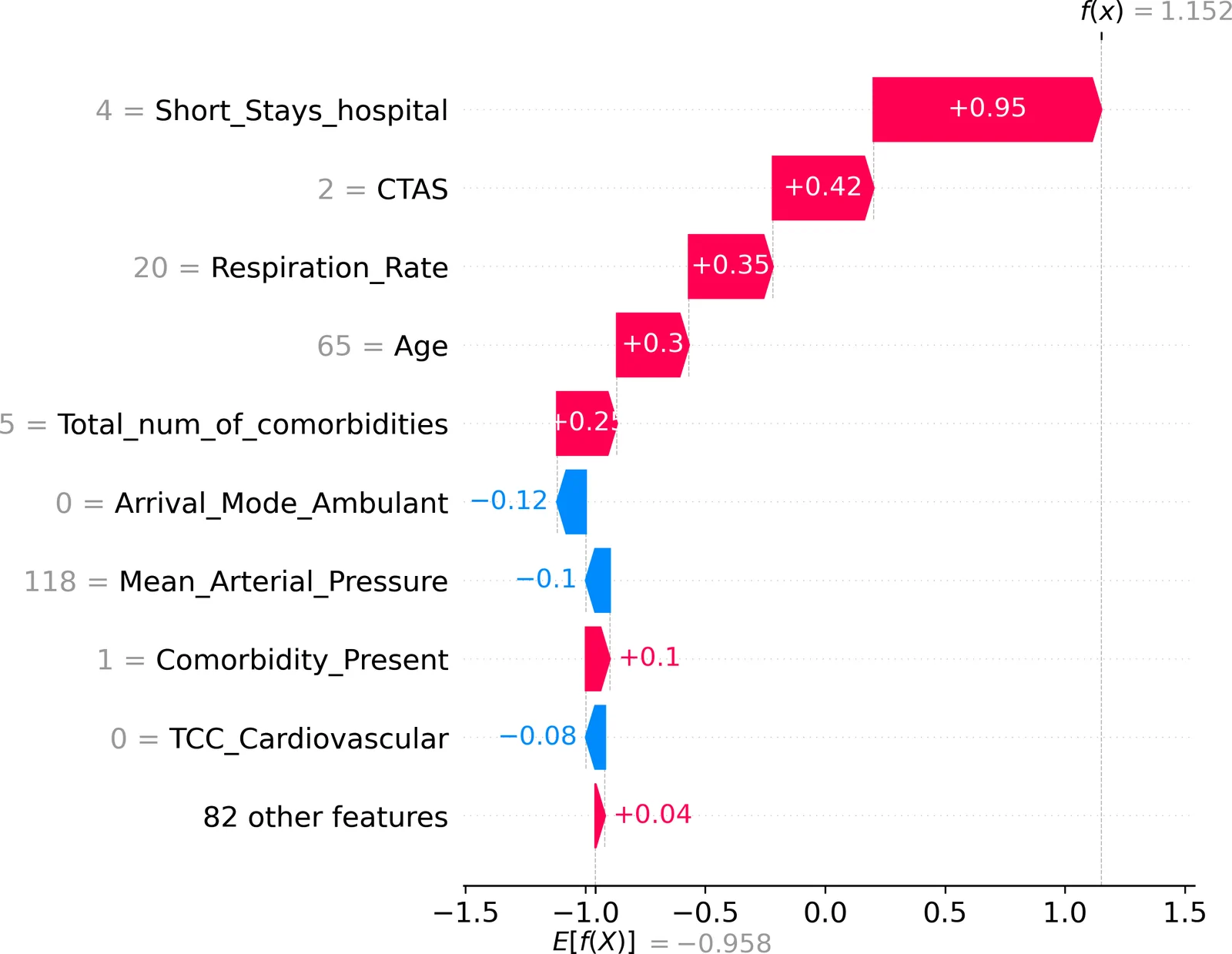
Shapley additive explanations waterfall plot illustrating feature contributions for a hypothetical individual patient prediction. CTAS: Canadian Triage and Acuity Scale; TCC: triage chief complaint.

The SHAP force plot (Figure 5) presents the same information in a horizontal format, clearly displaying the dynamics between features that increase or decrease the prediction [34]. These explainability tools support clinician trust by offering transparency into the model’s rationale, particularly in ambiguous or borderline cases.

**Figure 5.**
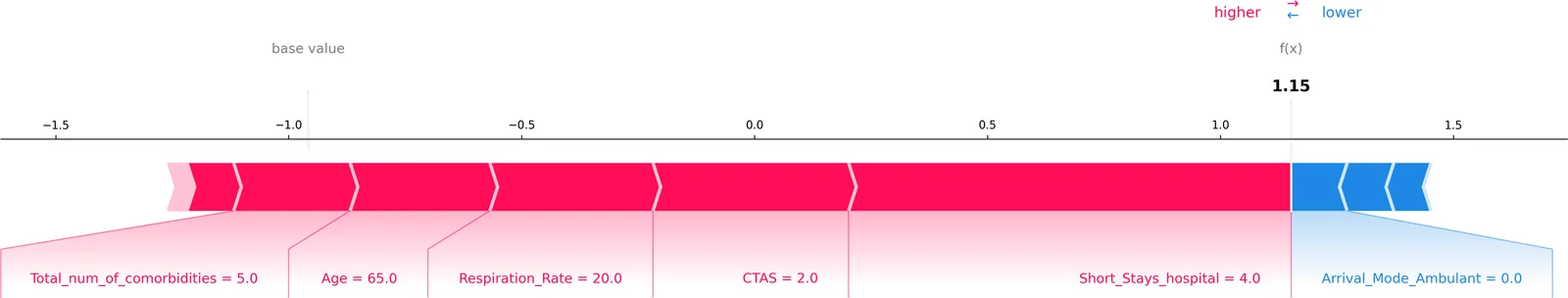
Shapley additive explanations force plot showing the prediction explanation for a hypothetical individual patient visit. CTAS: Canadian Triage and Acuity Scale.

An interesting insight emerged from the SHAP beeswarm plot concerning the frequency of ED visits in the past 12 months (Figure S2 in Multimedia Appendix 3). It was observed that patients in the high-frequency tail (eg, those with higher numbers of prior ED visits) were less likely to be admitted. This counterintuitive finding prompted further investigation. We hypothesized that the urgency of the presenting complaint, as measured by CTAS, might modify the relationship between visit frequency and admission.

As an exploratory, post hoc analysis intended for hypothesis generation, we built 3 LR models (Table 3): Model 1 excluded CTAS, Model 2 included CTAS, and Model 3 included an interaction term between CTAS and ED visit frequency. In Models 1 and 2, the main effect for ED visit frequency was positive and highly significant (0.0209 and 0.0210, respectively; *P*<.001), yielding an odds ratio of approximately 1.02 per additional visit. This positive sign indicates that across the bulk of the cohort, an increase in prior visits is associated with a slightly higher baseline likelihood of admission. Furthermore, the positive interaction term in Model 3 (0.0066) indicates that this linear association becomes stronger for patients with less urgent conditions (higher CTAS scores). However, this simple linear trend is heavily influenced by the vast majority of patients who have very few prior visits (mean 1.2 visits, SD 3.0). The heatmap (Figure 6) reveals the true, nonlinear complexity of this relationship. Over the range containing nearly the whole cohort (0 to 5 prior visits), admission rates consistently rise across CTAS levels; for example, CTAS 3 admission rates rise from 0.14 to 0.28 over this range. The descending trend observed in the SHAP analysis is isolated strictly to the high-frequency tail (12+ visits), where extreme frequent usage is associated with a significantly lower likelihood of admission. This visual drop at the extremes is partly driven by shrinking patient volumes as visit frequency increases.

**Table 3.** Logistic regression coefficients from 3 models analyzing ED^a^ visit frequency.

Variable	Model 1 (without CTAS^b^), coefficient (SE)	Model 2 (with CTAS), coefficient (SE)	Model 3 (with interaction), coefficient (SE)
Constant	−1.5858^c^ (0.004)	0.0836^c^ (0.012)	0.1089^c^ (0.013)
ED^a^ visit frequency	0.0209^c^ (0.001)	0.0210^c^ (0.001)	0.0018^c^ (0.003)
CTAS	—^d^	−0.5682^c^ (0.004)	−0.5769^c^ (0.004)
Interaction	—	—	0.0066^c^ (0.001)

a ED: emergency department.

b CTAS: Canadian Triage and Acuity Scale.

c *P*<.001.

d Not available.

**Figure 6.**
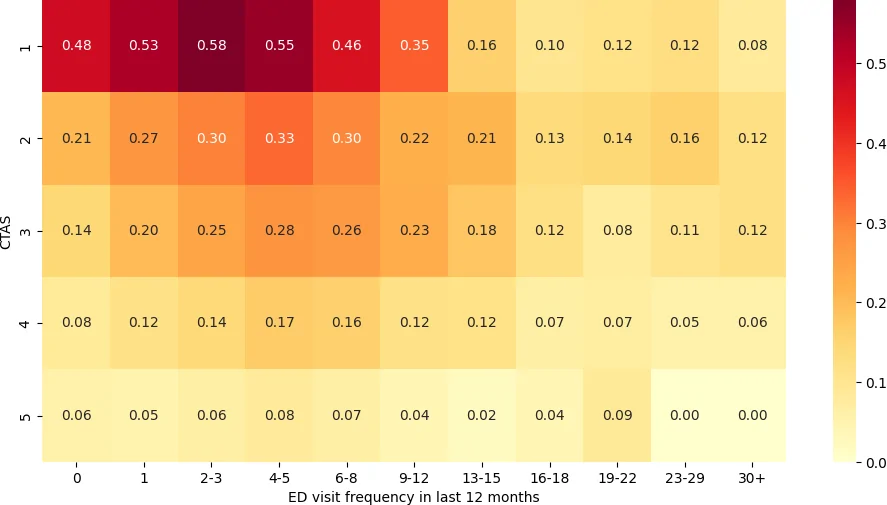
Heatmap of hospital admission rates by Canadian Triage and Acuity Scale (CTAS) and emergency department (ED) visit frequency.

### Subgroup Analysis

To evaluate the clinical applicability of our predictive models, we conducted stratified analyses by age (18‐39, 40‐64, 65 years and older), biological sex (male and female), and hospital site (MGH and RVH). Independent models were developed using the XGBoost algorithm for each subgroup using identical preprocessing and hyperparameter optimization protocols to isolate population-specific performance patterns while controlling for methodological variability.

Site-specific comparisons revealed divergent operational profiles despite both MGH and RVH being tertiary care centers. The model for MGH demonstrated higher accuracy (0.847 vs 0.766) and specificity (0.937 vs 0.756), while the model for RVH showed superior sensitivity (0.817 vs 0.438). These differences likely reflect variations in patient flow and case mix complexity. The model for MGH exhibited more definitive admissions (precision: 0.605 vs 0.404) and better-calibrated probabilities (log loss: 0.347 vs 0.482), suggesting distinct institutional dynamics influencing model performance.

Sex-stratified models exhibited clinically meaningful variations, with the model for female patients showing higher sensitivity (0.568 vs 0.457) but lower precision (0.525 vs 0.623). Both models maintained strong specificity (greater than 0.900) and comparable calibration (log loss: 0.335 female vs 0.353 male), suggesting balanced performance across sex groups despite different operating characteristics.

The age-stratified models demonstrated a clinically meaningful performance gradient, with accuracy declining from 0.914 (18‐39 years) to 0.698 (65 years and older), reflecting increasing clinical complexity across age groups. Younger adults (18‐39 years) exhibited higher specificity (0.961) but lower sensitivity (0.364). The 40‐64 years cohort showed a distinctive profile with the highest precision (0.650) despite reduced sensitivity (0.297). In contrast, the 65 years and older model exhibited a shift toward higher sensitivity (0.798) and lower specificity (0.653), and its elevated log loss (0.571) indicates a continuing need for probability calibration in this high-risk group. The complete performance metrics for all subgroup models are provided in Table 4.

**Table 4.** Performance metrics for the ML^a^ model across subgroups.

Feature and subgroups		N	Accuracy	AUC-ROC^b^ (95% CI)	Sensitivity (recall)	Specificity	PPV^c^ (precision)	Log loss
Site								
MGH^d^		231,705	0.8471	0.8497 (0.8454-0.8539)	0.4377	0.9371	0.6047	0.3469
RVH^e^		294,440	0.7664	0.8674 (0.8645-0.8717)	0.8167	0.7562	0.4042	0.4820
Sex								
Male		253,332	0.8413	0.8535 (0.8496-0.8575)	0.4566	0.9336	0.6227	0.3534
Female		272,767	0.8532	0.8599 (0.8559-0.8640)	0.5676	0.9057	0.5251	0.3354
Age (years)								
18‐39		188,805	0.9139	0.8507 (0.8396-0.8538)	0.3637	0.9612	0.4465	0.2415
40‐64		181,720	0.8682	0.8448 (0.8395-0.8502)	0.2972	0.9711	0.6499	0.3150
65 and older		155,620	0.6981	0.8046 (0.7995-0.8097)	0.7976	0.6529	0.5111	0.5707

a ML: machine learning.

b AUC-ROC: area under the receiver operating characteristic curve.

c PPV: positive predictive value.

d MGH: Montreal General Hospital.

e RVH: Royal Victoria Hospital.

### Sensitivity Analysis

To evaluate the stability of our model, we conducted 2 sensitivity analyses focusing on outcome definition and temporal data leakage. To assess the impact of these factors, we compared the original model to 2 sensitivity models. In the original model, the positive class included “admitted” only, and the negative class included “discharged” only. All other dispositions were ignored. The data were partitioned using random stratified sampling across the entire 8-year dataset. In sensitivity model 1 (outcome definition), the positive class included “admitted,” “deceased,” and “transferred.” The negative class included the remaining disposition categories. Similar to the original model, the data were partitioned using random stratified sampling across the entire 8-year dataset. Finally, in sensitivity model 2 (temporal split), the outcome definitions were identical to those of the original model. The data were partitioned chronologically, with the training set spanning 2015 to 2020, the validation set covering 2021, and the testing set restricted to 2022.

All models were trained using identical modeling procedures and feature sets as the original XGBoost model. Performance metrics for all 3 models are presented in Table 5.

**Table 5.** Performance comparison between the original model and the sensitivity analysis models.

Metric	Original model	Sensitivity model 1	Sensitivity model 2
AUC-ROC^a^	0.8608 (0.8580-0.8635)	0.8702 (0.8677-0.8726)	0.8531 (0.8494-0.8568)
AUC-PR^b^	0.5863	0.6092	0.5909
Accuracy	0.8259	0.8345	0.7867
Sensitivity (recall)	0.6691	0.6763	0.7493
Specificity	0.8589	0.8673	0.7953
PPV^c^ (precision)	0.4991	0.5140	0.4544
*F*_1_-score	0.5717	0.5841	0.5657
Log loss	0.3792	0.3639	0.4473

a AUC-ROC: area under the receiver operating characteristic curve.

b AUC-PR: area under the precision-recall curve.

c PPV: positive predictive value.

For Sensitivity Model 1, including the deceased in the admitted class was guided by clinical reasoning. These patients usually arrive in critical condition and likely would have been admitted had they survived. This pattern is reflected in the data, where the mean CTAS score among deceased patients was 1.7 (SD 0.94), showing high acuity at triage. The classification of transferred patients posed a challenge because no follow-up data were available. Emergency physicians at the MUHC noted that transfers between sites usually occur with the plan to admit the patient at the receiving hospital. Patients who are expected to be discharged are generally not transferred. This aligns with the SHAP feature importance plot, which shows higher admission likelihood for patients arriving from another hospital. Based on this reasoning, transferred patients were grouped with admitted individuals. LWBS patients were placed in the discharged class. These individuals were stable enough to leave on their own, and if they had stayed, they would most likely have been discharged. This makes their placement in the discharged group clinically appropriate.

Although performance improved slightly after adding these patients to the admitted class, the differences between the original model and Sensitivity Model 1 were not large. The sensitivity model showed modest gains in AUC-ROC, AUC-PR, accuracy, sensitivity, specificity, precision, and *F*_1_-score, along with a lower log loss. These results indicate that broadening the outcome definition did not meaningfully change the model’s behavior. Instead, the model remained stable, suggesting that its predictions are shaped by the overall clinical patterns in the data rather than by how these additional disposition categories are assigned.

For Sensitivity Model 2, cross-validation was performed using the TimeSeriesSplit module from the scikit-learn package [27] to maintain temporal integrity during hyperparameter tuning. The temporal split resulted in a slight decrease in overall discrimination, with the AUC-ROC shifting from 0.8608 to 0.8531. Sensitivity increased to 0.7493, while the specificity and precision dropped. Log loss also increased to 0.4473. This shift is expected and reflects concept drift caused by the COVID-19 pandemic. Hospital admission criteria likely became stricter in 2022 due to capacity constraints. Because the temporal model learned pre-2021 admission rules, it naturally overestimated the need for beds under the new protocols, leading to higher sensitivity and lower precision. Testing exclusively on 2022 isolates the evaluation to a period of extreme operational disruption. Therefore, the original random sampling approach provides a more balanced evaluation of the model’s general performance. It ensures a proportional representation of prepandemic, pandemic, and postpandemic clinical patterns. This prevents the overall assessment from being skewed by the atypical systemic constraints of any single period.

### Threshold Decision Analysis

While a standard probabilistic threshold of 0.5 is commonly used in binary classification, it implies equal weight between false positives (incorrectly predicting admission) and false negatives (missing an admission). In the context of ED bed management, however, operational requirements may necessitate prioritizing the PPV to ensure that when a bed is reserved, it is actually used. To address this, we analyzed the stability of the model and the characteristics of the population across a range of decision thresholds by examining patients whose predicted outcome changed from “admit” to “not admit” as the threshold increased.

The analysis revealed that as the threshold rises, the model effectively gains true negatives (correct exclusions) at the expense of introducing false negatives (missed admissions). As illustrated in the top panel of Figure 7, the absolute volume of reclassified patients remains high across the initial threshold steps, peaking at 1718 patients (0.55 to 0.60) before steadily declining to 1489 patients in the highest observed step (0.75 to 0.80). Between thresholds of 0.50 and 0.55, 72% (1212/1680) of the patients who flipped were true negatives, and between 0.55 and 0.60, this figure was 68%. This indicates a meaningful improvement in PPV with a relatively lower loss in sensitivity. A crossover point occurs much later between 0.75 and 0.80, where the proportion of missed admissions (orange bars) becomes dominant, reaching 53%, representing a sharp decline in model sensitivity.

**Figure 7.**
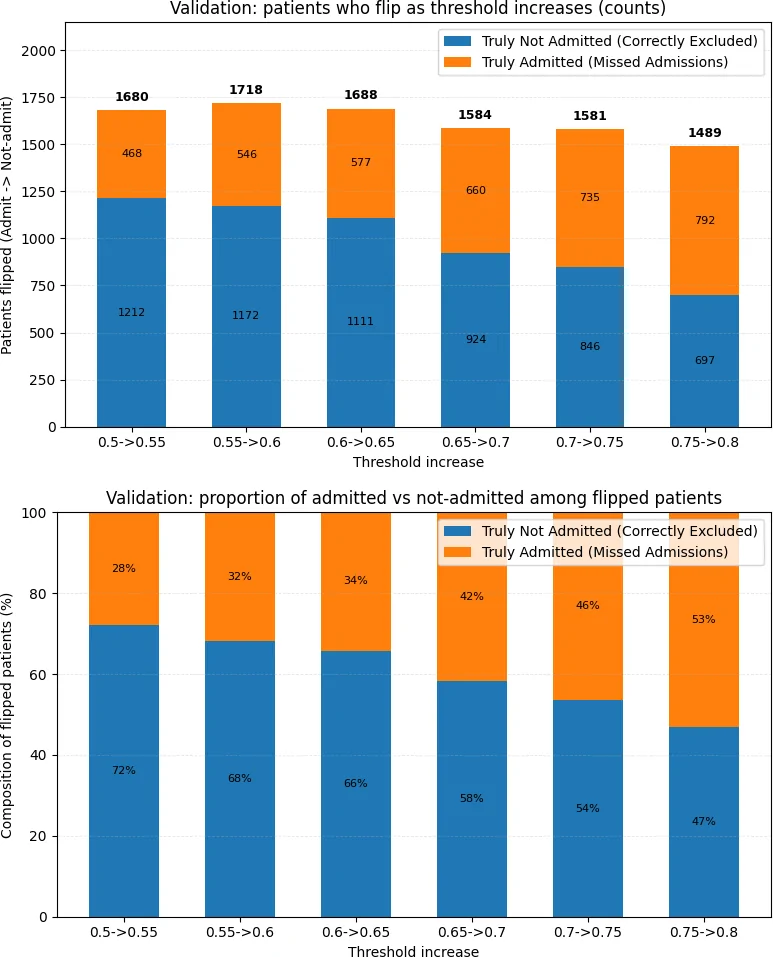
Threshold flip behavior analysis. The top panel displays the absolute count of patients whose classification flipped from “admit” to “not admit” at each threshold increment. The bottom panel illustrates the proportional composition of these flipped patients, demonstrating that at thresholds >0.75, the majority of excluded patients are true admissions (missed). Evaluated on the validation set.

To determine if these flips were driven by specific patient demographics or acuity levels, the clinical profiles of flipped patients were evaluated across threshold steps (Figure 8). The median age for both correctly excluded and missed patients remained stable across all threshold increments, fluctuating only slightly between 62 and 72 years. Similarly, the distribution of CTAS scores showed no significant deviation; patients in acuity levels 2 and 3 consistently accounted for the majority of flipped cases for both true negatives and missed admissions. This stability suggests that threshold adjustments reflect a global shift in model conservatism rather than a bias against specific age groups or acuity levels.

**Figure 8.**
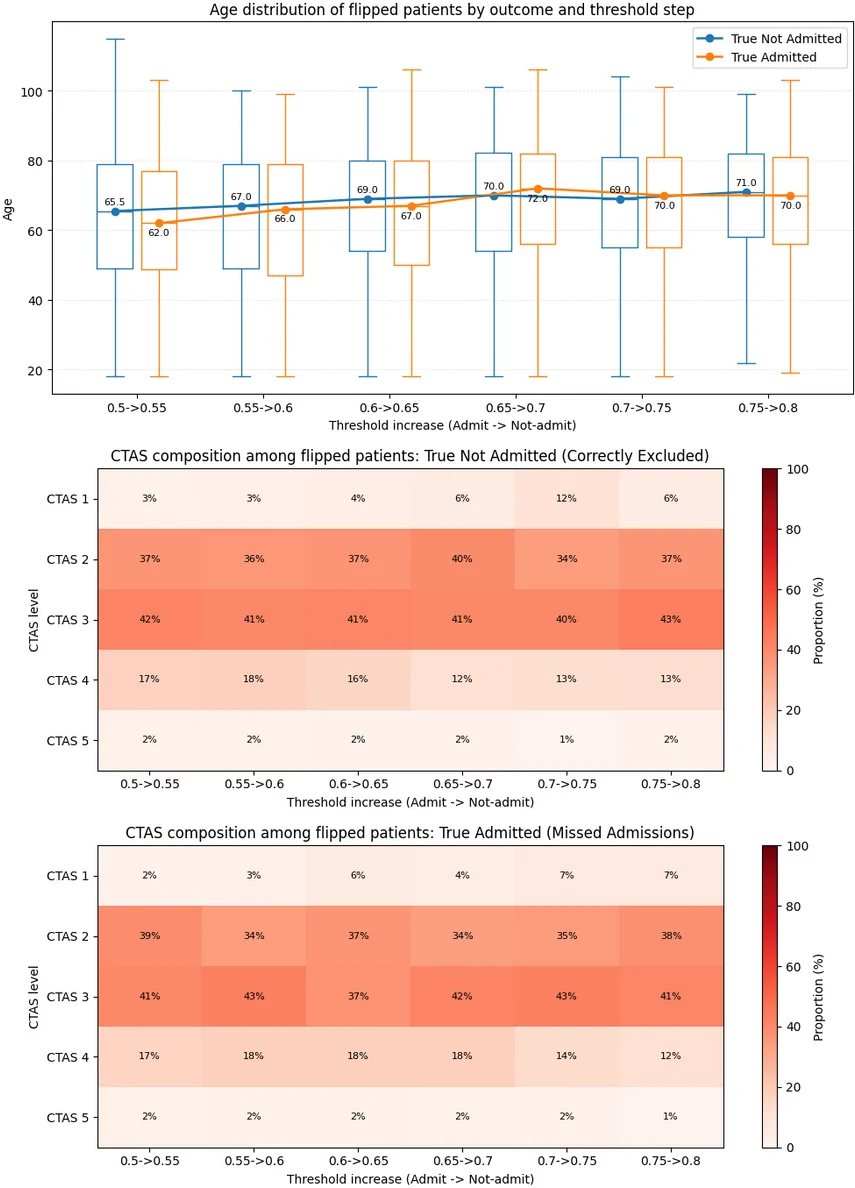
Clinical profile of flipped patients by age and acuity. The top box plot shows stable age distributions for both correctly excluded (blue) and missed admitted (orange) patients across threshold steps. The bottom heatmaps display the Canadian Triage and Acuity Scale (CTAS) level composition, indicating that the distribution of acuity remains consistent regardless of the threshold used. Evaluated on the validation set.

Finally, the trade-off between classification metrics was evaluated to identify an optimal operational range. Figure 9 displays the Sensitivity, PPV, and *F*-scores as a function of the decision threshold. While sensitivity naturally declines as the threshold increases, precision (PPV) improves. The *F*_0.5_-score, which prioritizes PPV over sensitivity, peaks at a threshold of approximately 0.72. Given that age and CTAS profiles remain stable, a threshold in the 0.70 to 0.75 range appears to offer the most operationally balanced trade-off, minimizing false positives by accepting reduced sensitivity in exchange for a substantial gain in precision. This optimal decision threshold range was determined exclusively using the validation set to preserve the strict independence of the held-out test set.

**Figure 9.**
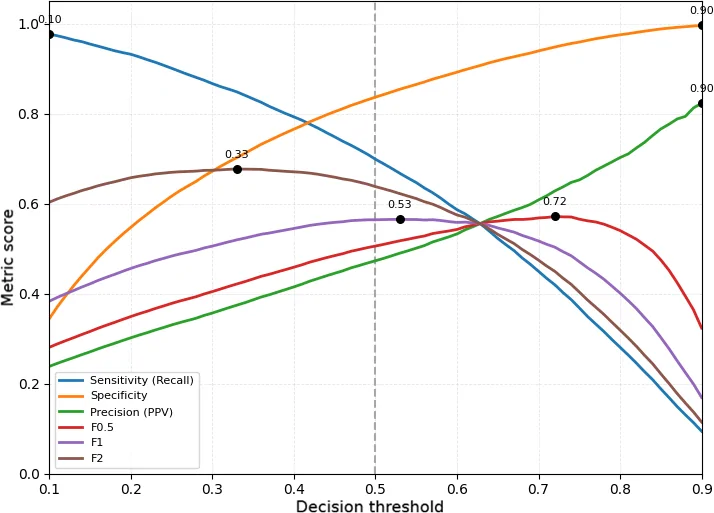
Classification metrics as a function of the decision threshold. The curves illustrate the trade-off between sensitivity (recall) and precision (positive predictive value). The *F*_0.5_-score peaks near a threshold of 0.72, suggesting an optimal balance for identifying admissions with higher confidence. Evaluated on the validation set. PPV: positive predictive value.

## Discussion

### Principal Findings

Hospital admission following ED visit was accurately predicted by the XGBoost model, which demonstrated strong performance (AUC-ROC=0.8608; 95% CI 0.8580‐0.8635). The model achieved high sensitivity (0.6691) and specificity (0.8589), indicating good discriminative ability for identifying patients who require hospitalization. However, the moderate precision (0.4991) indicates a tendency toward false-positive predictions.

Feature importance analysis revealed that clinical factors such as the patient’s age, triage acuity level, and vital sign measurements, particularly pulse rate and temperature, were among the most influential predictors. This finding underscores the importance of objective clinical assessments in determining admission decisions. In addition, health care system characteristics, particularly prior hospitalization frequency and patient origin being a hospital, were also significant predictors. An interaction was observed between ED visit frequency and CTAS, whereby the association between frequent ED use and likelihood of admission varied across CTAS levels, indicating that clinical urgency modifies the relationship between prior usage and disposition. This highlights the role of patients’ medical history and care trajectories in influencing disposition outcomes.

In contrast, several features demonstrated limited predictive value. These included all temporal variables (eg, hour of day, day of the month, and season of arrival), the number of ED arrivals in the 60 minutes preceding the patient’s arrival, and the number of prior ED visits within the past 12 months. While prior ED visits had low global importance, their complex interaction with clinical urgency warranted specific attention. The SHAP importance of the ED crowding metric (Arrivals_Past_60_Min) was minimal compared to core clinical features like age, CTAS, and vital signs. This is an important distinction because it shows the model avoids operational circularity. If this crowding variable had been highly predictive, the model might have learned to predict access block and bed availability rather than a patient’s true clinical need. Certain vital signs, including the derived BP metrics (PP, MAP, and BP ratio), GCS, and glucose level, also contributed minimally. Among comorbidities, only aggregate indicators such as the total number of comorbidities and the presence of at least one were meaningfully associated with admission predictions. Interestingly, individual comorbidities ranked low in predictive importance, possibly because their effects on admission decisions are indirectly captured by more immediate clinical indicators such as vital signs, acuity scores, or recent hospitalization history. With respect to TCCs, categories such as gastrointestinal, musculoskeletal, trauma, and ENT and ophthalmological were more influential, while dermatological, infectious, respiratory, and genitourinary complaints showed lower importance in the model.

Sensitivity analyses showed that alternative outcome definitions and chronological data splits maintained strong discrimination (AUC-ROC>0.85). While pandemic-driven concept drift shifted calibration, the overall model stability highlights the importance of clearly specifying evaluation periods and disposition categories in predictive modeling.

An operational threshold evaluation demonstrated that the default 0.5 cut-off is not fixed. Guided by the *F*_0.5_-score, shifting the decision boundary to the 0.70 to 0.75 range significantly improved PPV. This adjustment reduces false bed reservations by prioritizing a gain in precision in exchange for reduced sensitivity. A “flip analysis” suggested this shift does not disproportionately impact specific age or acuity profiles. Consequently, the model offers operational flexibility, allowing administrators to tune performance for real-time capacity without disproportionately affecting specific patient subgroups.

Overall, these findings suggest that ML models using only tabular data from data warehouses, including demographic, clinical, and system-level variables, can provide valuable, interpretable decision support for predicting hospital admissions from the ED.

### Comparison With Previous Studies

Several studies have explored ML models for predicting hospital admission from the ED using data available at or shortly after triage. While reported discrimination performance has generally been strong, with AUC-ROC values ranging from approximately 0.82 to 0.92, these studies differ considerably in methodology and scope. Hong et al [37] and De Hond et al [38] included features beyond the initial triage window, such as patient history or data collected after arrival, which limits the applicability of their models for real-time decision-making. In contrast, Araz et al [39] and Cusidó et al [40] focused strictly on data available at triage, making their approaches more relevant to early prediction. However, these studies relied on a narrow set of input features, typically limited to demographics, vital signs, chief complaint, and acuity scores.

Across all 4 studies, model evaluation centered almost exclusively on discrimination metrics such as AUC-ROC, with little attention to calibration or other clinically relevant metrics, raising concerns about how well predicted probabilities reflect actual outcomes or how useful the models would be in practice. This is a notable gap, as strong discrimination does not necessarily imply that predictions are reliable or actionable.

Feature selection in prior studies was also constrained. For instance, Cusidó et al [40] found that just 3 variables contributed the majority of predictive power, while Hong et al [37] showed that a reduced model with a few dominant features performed nearly as well as one with hundreds of variables. However, none of the studies incorporated system-level indicators such as ED crowding metrics or detailed comorbidity profiles, which could improve operational relevance.

Missing data handling represented another limitation. None of the reviewed studies [37-40] discussed whether missingness was random or addressed MNAR scenarios. Imputation strategies were not described, leaving open the risk of bias and limiting the generalizability of findings in real-world settings where data are often incomplete in systematic ways.

Interpretability and explainability were minimally addressed. Although some studies reported feature importance [37,40], none used model-agnostic methods such as SHAP to generate global explanations. Without these tools, model predictions remain opaque to clinicians, limiting their trust and clinical uptake. Furthermore, subgroup performance was not adequately examined. For example, while De Hond et al [38] used a multicenter dataset, they did not assess variation in model accuracy by age, sex, or site. The absence of fairness evaluation leaves the potential for unrecognized bias.

Finally, sensitivity analyses were either lacking or narrowly defined. Most studies did not assess how model performance changes when excluding ambiguous disposition categories such as transfers, in-ED deaths, or patients who left without being seen by a physician. Understanding the validity of models under such exclusions is essential for ensuring generalizability and implementation viability.

A recent systematic review by Williams et al [41] evaluated 31 ML models designed to predict ED admissions using triage data. While the top-performing algorithms achieved AUC-ROC scores ranging from 0.81 to 0.93, the review highlighted several critical shortcomings across the broader literature. Most notably, many of the studies exhibited a high or unclear risk of bias alongside significant applicability concerns. These issues largely stemmed from opaque model reporting, the improper handling of missing data (such as excluding incomplete patient records entirely), and a failure to define clear clinical disposition outcomes.

Additionally, while prior studies span various countries, neither the existing literature nor our current study accounts for how cultural dimensions influence health care-seeking behavior. According to the framework by Hofstede et al [42], human behavior and cultural norms vary widely across societies. Differences in how populations tolerate health-related uncertainty or perceive medical authority can dictate whether patients use EDs for minor issues or delay visits until their condition is critical. These cultural behaviors shape the baseline data that ML models learn from. Consequently, the inability to account for these cultural variables remains a shared limitation across the field, making direct international comparisons of model performance challenging.

In summary, this study addresses these gaps by developing calibrated, interpretable models based strictly on triage-time data, incorporating a broader feature set, and evaluating subgroup performance and sensitivity through model-agnostic explainability techniques to enhance real-world applicability.

### Strengths and Limitations

This study possesses several key strengths that enhance its potential for clinical impact. First, it is based on a large, real-world dataset comprising 526,145 ED visits collected over 8 years from 2 academic tertiary care hospitals within the MUHC network. The inclusion of 2 distinct academic hospitals within the MUHC network (MGH and RVH) improves the diversity of the study population by capturing variation in patient demographics and case mix while maintaining consistency in clinical and administrative practices. Second, the model development prioritized features readily available at or right after triage, enabling predictions in near real-time and facilitating future clinical integration. Unlike studies that depend heavily on laboratory or imaging results, thereby delaying prediction [37], this approach aligns with the urgent decision-making context characteristic of emergency care. Third, the models demonstrated strong discriminative performance, with XGBoost achieving an AUC-ROC of 0.8608 (95% CI 0.8580‐0.8635), comparable to or exceeding prior leading work in ED predictive analytics.

The study’s emphasis on explainability further distinguishes it. By leveraging SHAP values [34], we provided feature-level attributions that enhance transparency and support clinician trust, which is an essential requirement for responsible ML in health care [43]. Independent subgroup-specific models were developed across hospital sites, sex, and age categories to evaluate whether tailoring algorithms to distinct clinical subpopulations maintains strong predictive performance. Furthermore, strict methodological standards were used to prevent data leakage [23], including isolated preprocessing pipelines for training, validation, and testing sets. Bayesian optimization was used for hyperparameter tuning to maximize generalizability while mitigating overfitting risks [36]. Finally, the threshold flip analysis demonstrated operational stability, confirming that adjusting the decision boundary to manage bed capacity does not inadvertently skew predictions against older or higher-acuity patients, a critical requirement for safe clinical deployment. To contextualize this operationally, the dataset’s 91,363 total admissions over 2,922 days average 31 admissions daily. At the 0.50 threshold (69.9% sensitivity), the miss rate translates to approximately 9 missed early bed allocations per day. Shifting the threshold to 0.70 (45% sensitivity) increases this to roughly 17 daily misses. Crucially, because this is an early bed management tool rather than a diagnostic one, false negatives do not lead to unsafe discharges. Patients still receive standard physician evaluations, meaning missed predictions represent lost opportunities for early bed preparation rather than clinical safety risks.

Another methodological strength of this study was the inclusion of a sensitivity analysis to assess the effect of alternative outcome definitions on model performance. By reclassifying deceased patients as admitted and testing models with and without transferred patients, we demonstrated that while AUC-ROC remained stable, other metrics such as sensitivity, specificity, and precision varied. This underscores the importance of clearly defined and consistently recorded outcomes for training and evaluating predictive models in emergency care.

Nonetheless, several limitations must be acknowledged. First, our dataset is restricted to 2 large, urban academic hospitals within a publicly funded health care system. While our models may generalize to similar tertiary care EDs, they may not translate to rural community hospitals operating under different resource constraints. Furthermore, the findings may not generalize to distinct systemic or cultural settings. Systemically, private or insurance-based health care systems operate with different financial incentives, triage protocols, and admission thresholds than Canada’s publicly funded model. Culturally, as highlighted by Hofstede et al [42], human behavior regarding health care usage varies globally. Differences in cultural norms can significantly alter ED presentation patterns and case mix. External validation across these varying environments is essential before clinical deployment. Second, the retrospective nature of the dataset carries inherent risks of biases due to historical documentation practices, evolving triage protocols, and missing data patterns. While missingness was explicitly handled and modeled where appropriate, retrospective designs cannot fully eliminate such biases. Third, the study focused exclusively on structured data elements, omitting unstructured information from clinical notes, which has been shown to improve predictive performance when incorporated through natural language processing techniques [44].

Moreover, while SHAP improves interpretability, tree-based models such as XGBoost can still obscure complex feature interactions, and their interpretability may not match that of traditional statistical models like LR [45]. Furthermore, not all critical determinants of disposition decisions, including social circumstances, caregiver support, and system-level operational pressures, were available in the dataset [46]. Finally, while internal validation on a held-out dataset was performed, real-world prospective validation remains necessary. Implementation in clinical practice may reveal workflow integration barriers, clinician override behavior, and issues of data timeliness that could affect performance [47]. Finally, as hospital admission decisions are influenced by operational pressures such as bed availability and staffing, the model may reflect both clinical acuity and systemic capacity constraints [48,49].

Our subgroup analysis revealed that model calibration was noticeably worse for patients aged 65 and older compared to younger cohorts. This carries important clinical safety implications since older adults represent a vulnerable population with the highest baseline admission rates. The poorer calibration likely stems from complex presentations and multimorbidity that standard triage variables do not fully capture. To ensure safe clinical deployment, future implementations must address this gap through age-specific recalibration techniques, such as post-hoc Platt scaling [50], or by developing dedicated geriatric models.

Additionally, we split the data at the encounter level rather than the patient level. This introduces a potential risk of within-patient data leakage for static variables if a patient appears in both the training and test sets. However, about 64% (171,837/268,496) of patients in our cohort had only a single visit, limiting this risk for most of the dataset. For returning patients, acute clinical variables like vital signs and triage acuity change significantly between visits. Furthermore, by explicitly including “ED Visits in the Past 12 Months” as a feature, the model learns generalized patterns of frequent usage rather than memorizing individual profiles. The dataset was partitioned using random stratified sampling instead of a temporal holdout. A chronological split reserving the final years for testing would isolate the evaluation entirely to the COVID-19 pandemic. Testing exclusively under such extreme operational disruptions would skew the assessment. Random sampling ensured proportional representation of prepandemic, pandemic, and postpandemic patterns. Future prospective validation is needed to confirm performance in standard clinical workflows.

Furthermore, regarding historical usage features, our dataset is restricted to the MUHC network. Consequently, it cannot account for prior health care usage at external hospital networks due to siloed databases. However, because clinical algorithms must currently operate within these same fragmented data environments, our results provide a realistic estimate of baseline operational performance. Future implementations could likely be enhanced as regional data sharing and interoperability improve.

Another methodological strength of this study was the use of sensitivity analyses to assess model stability against alternative outcome definitions and temporal data splits. By reclassifying deceased and transferred patients, we demonstrated that while overall discrimination remained stable, metrics like sensitivity and precision varied. This underscores the importance of clearly defined outcomes in emergency care. Applying a strict chronological train-test split proved that while the COVID-19 pandemic altered admission thresholds and affected model calibration, the core predictive features remained highly stable over an 8-year period.

In summary, while the findings demonstrate that ML models can accurately predict hospital admission status using triage data alone, this study’s limitations highlight important considerations for generalization, prospective testing, and integration with broader clinical decision-making frameworks.

### Conclusions

This study developed ML models to predict hospital admissions early during ED visits, using only data available at triage. The models achieved strong predictive performance while maintaining simplicity and clinical interpretability. By applying explainable AI techniques, the study enhanced transparency and provided insights into the most influential predictors. Compared to previous research, our models demonstrated equivalent or better explainable performance at the point of triage, enabling real-time implementation and supporting clinical interpretation and trust. Furthermore, the model demonstrates operational stability, allowing hospitals to adjust decision thresholds to manage bed capacity while maintaining stable performance across age and higher-acuity subgroups. Although the findings are promising, further external validation and prospective evaluations are necessary to confirm generalizability and impact. Overall, the study underscores the potential of ML to support early decision-making and optimize ED operations.

## Supplementary material

10.2196/98273 Multimedia Appendix 1 TRIPOD+AI checklist for the abstract.

10.2196/98273 Multimedia Appendix 2 TRIPOD+AI reporting checklist for the main paper

10.2196/98273 Multimedia Appendix 3 Supplementary tables and figures detailing dataset feature distributions, model hyperparameter configurations, and extended performance metrics.

## Abbreviations

ANN: artificial neural network
AUC-PR: area under the precision-recall curve
AUC-ROC: area under the receiver operating characteristic curve
BP: blood pressure
CatBoost: categorical boosting
CTAS: Canadian Triage and Acuity Scale
DBP: diastolic blood pressure
DT: decision tree
ED: emergency department
EHR: electronic health record
GCS: Glasgow Coma Scale
LGBM: light gradient boosting machine
LR: logistic regression
LWBS: left without being seen
MAP: mean arterial pressure
MGH: Montreal General Hospital
ML: machine learning
MLP: multilayer perceptron
MNAR: missing not at random
MUHC: McGill University Health Centre
NPV: negative predictive value
PPV: positive predictive value
RF: random forest
RVH: Royal Victoria Hospital
SBP: systolic blood pressure
SHAP: Shapley additive explanations
SMOTE-Tomek: Synthetic Minority Oversampling Technique with Tomek Links
TCC: triage chief complaint
TRIPOD: Transparent Reporting of a Multivariable Prediction Model for Individual Prognosis or Diagnosis
XGBoost: extreme gradient boosting
