# Supplementary material for: Explainable Machine Learning for Predicting Hospital Admission at Emergency Department Triage: Retrospective Cohort Study
Source: JMIR Med Inform. 2026 Nov 10;14:e98273. doi: 10.2196/98273 (PMC13750794; doi:10.2196/98273)
Supplement: Supplementary file 2 [file medinform-v14-e98273-s003.pdf]

Multimedia Appendix 1. Supplementary Tables and Figures

Table S1. Distribution of demographic features in the study population stratified by dataset.

Features	Overall	Training Set	Validation Set	Test Set
Sex				
Female	272,767 (51.8)	190,626 (51.8)	27,215 (52.0)	54,926 (51.9)
Unknown	46 (0.008)	36 (0.009)	3 (0.006)	7 (0.007)
Age	50.9 (21.1)	50.9 (21.1)	50.9 (21.1)	51 (21.0)
Arrival Mode				
Walk-in	368,769 (70.0)	258,027 (70.1)	36,545 (70.2)	74,197 (70.2)
Ambulance	148,879 (28.3)	104,345 (28.3)	14,689 (28.2)	29,845 (28.2)
Patient Origin				
Residence	376,414 (77.6)	263,586 (77.6)	37,189 (77.4)	75,639 (77.5)
Hospital	20,190 (4.2)	14,031 (4.1)	1,987 (4.1)	4,172 (4.3)
Clinic	18,494 (3.8)	12,984 (3.8)	1,867 (3.9)	3,643 (3.7)
Workplace	14,614 (3.0)	10,171 (3.0)	1,504 (3.1)	2,939 (3.0)
Without a home	16,957 (3.5)	11,879 (3.5)	1,644 (3.4)	3,434 (3.5)
Site				
RVH	294,440 (55.9)	206,087 (56.0)	29,127 (55.9)	59,226 (56.0)
MGH	231,705 (44.1)	162,214 (44.0)	22,961 (44.1)	46,530 (44.0)

Note. RVH = Royal Victoria Hospital, MGH = Montreal General Hospital. Values are *n* (%) for categorical variables and mean (SD) for continuous variables. Percentages may not total 100 due to rounding.

Table S2. Distribution of triage features in the study population stratified by dataset.

Features	Overall	Training Set	Validation Set	Test Set
CTAS				
1	13,505 (2.6)	9,434 (2.6)	1,345 (2.6)	2,726 (2.6)
2	133,098 (25.3)	93,122 (25.3)	13,110 (25.2)	26,866 (25.4)
3	205,398 (39.0)	143,725 (39.0)	20,341 (39.0)	41,332 (39.1)
4	136,813 (26.0)	95,859 (26.0)	13,613 (26.1)	27,341 (25.9)
5	37,329 (7.1)	26,160 (7.1)	3,679 (7.1)	7,490 (7.1)
Triage Chief Complaint				
Gastrointestinal	81,146 (15.4)	57,111 (15.5)	7,960 (15.3)	16,075 (15.2)
Musculoskeletal	73,221 (13.9)	51,373 (14.0)	7,186 (13.8)	14,662 (13.9)
Cardiovascular	61,960 (11.8)	43,303 (11.8)	6,184 (11.9)	12,473 (11.8)
Trauma	49,495 (9.4)	34,594 (9.4)	4,933 (9.5)	9,968 (9.4)
Neurological	41,012 (7.8)	28,648 (7.8)	4,187 (8.0)	8,177 (7.7)
Oxygen Saturation	97.6 (2.4)	97.6 (2.4)	97.6 (2.4)	97.6 (2.3)
Temperature	36.8 (0.7)	36.8 (0.7)	36.8 (0.7)	36.8 (0.7)
Respiration Rate	18.1 (2.7)	18.1 (2.8)	18.1 (2.7)	18.1 (2.8)
Pulse Rate	85.8 (18.0)	85.8 (18.0)	85.7 (18.1)	85.8 (18.1)
Diastolic BP	78.8 (12.1)	78.8 (12.1)	78.8 (12)	78.8 (12.1)
Systolic BP	132 (22.4)	132 (22.4)	132.1 (22.5)	132 (22.4)
Glasgow Coma Scale	14.7 (1.5)	14.7 (1.5)	14.7 (1.7)	14.7 (1.7)
Glucose Level	8.2 (4.2)	8.2 (4.2)	8.2 (4.2)	8.2 (4.2)
Pain Score	5.4 (3.1)	5.4 (3.1)	5.4 (3.1)	5.4 (3.1)
Mean Arterial Pressure	96.5 (13.9)	96.6 (13.9)	96.5 (13.9)	96.5 (13.9)
Pulse Pressure	53.3 (17.7)	53.3 (17.7)	53.3 (17.9)	53.3 (17.7)
BP Ratio	1.7 (0.3)	1.7 (0.3)	1.7 (0.5)	1.7 (0.3)
Missing Vital-Signs Count	2.3 (1.5)	2.3 (1.5)	2.3 (1.5)	2.3 (1.5)

Note. CTAS = Canadian Triage and Acuity Scale, BP = Blood Pressure. Values are *n* (%) for categorical variables and mean (SD) for continuous variables. Percentages may not total 100 due to rounding.

Table S3. Distribution of temporal features, patient history, and system factors in the study population stratified by dataset.

Features	Overall	Training Set	Validation Set	Test Set
**Temporal**				
Day of Month	15.7 (8.8)	15.7 (8.8)	15.7 (8.8)	15.8 (8.8)
Month of Arrival	6.5 (3.4)	6.5 (3.4)	6.6 (3.4)	6.5 (3.4)
Hour of Arrival	12.8 (5.7)	12.8 (5.7)	12.8 (5.7)	12.8 (5.7)
Day of Week	2.9 (1.9)	2.9 (2.0)	2.9 (2.0)	2.9 (2.0)
Is Weekend (Yes)	133,679 (25.4)	93,351 (25.3)	13,221 (25.4)	27,107 (25.6)
Season of Arrival				
Winter	127,336 (24.2)	88,947 (24.2)	12,676 (24.3)	25,713 (24.3)
Spring	131,027 (24.9)	91,754 (24.9)	12,860 (24.7)	26,413 (25.0)
Summer	134,796 (25.6)	94,440 (25.6)	13,363 (25.7)	26,993 (25.5)
Autumn	132,986 (25.3)	93,160 (25.3)	13,189 (25.3)	26,637 (24.9)
**Patient History**				
ED Visits-				
Past 12 Months	1.2 (3.0)	1.2 (3.0)	1.2 (3.0)	1.2 (3.0)
Short Stays Hospital	0.7 (1.9)	0.7 (1.9)	0.7 (1.9)	0.7 (2.0)
Day Surgeries Hospital	0.2 (0.6)	0.2 (0.6)	0.2 (0.6)	0.1 (0.6)
**System Factor**				
Arrivals Past 60 Min	5.7 (3.3)	5.7 (3.3)	5.7 (3.3)	5.7 (3.3)

Note. Values are *n* (%) for categorical variables and mean (SD) for continuous variables. Percentages may not total 100 due to rounding.

Table S4. Distribution of comorbidities in the study population stratified by dataset.

Features	Overall	Training Set	Validation Set	Test Set
Comorbidity Present (Yes)	136,879 (26.0)	95,909 (26.0)	13,536 (26.0)	27,434 (26.0)
Total Comorbidities-				
Present	0.8 (1.7)	0.8 (1.7)	0.8 (1.7)	0.8 (1.7)
Hypertension	85,964 (16.3)	60,246 (16.3)	8,487 (16.3)	17,231 (16.3)
Arrhythmias	2,031 (0.4)	1,395 (0.4)	199 (0.4)	437 (0.4)
Renal Disease	24,886 (4.7)	17,516 (4.8)	2,451 (4.7)	4,919 (4.7)
Mild Liver Disease	14,610 (2.8)	10,294 (2.8)	1,401 (2.7)	2,915 (2.8)
Moderate or Severe-				
Liver Disease	5,208 (1.0)	3,685 (1.0)	505 (1.0)	1,018 (1.0)

**Features**	**Overall**	**Training Set**	**Validation Set**	**Test Set**
Diabetes Mellitus-				
without Complications	42,473 (8.1)	29,781 (8.1)	4,202 (8.1)	8,490 (8.0)
Diabetes Mellitus-				
with Complications	9,168 (1.7)	6,446 (1.8)	904 (1.7)	1,818 (1.7)
Chronic Pulmonary Disease	38,678 (7.4)	26,955 (7.3)	3,856 (7.4)	7,867 (7.4)
Congestive Heart Failure	23,397 (4.4)	16,427 (4.5)	2,318 (4.5)	4,652 (4.4)
Peripheral Vascular Disease	15,682 (3.0)	10,990 (3.0)	1,528 (3.0)	3,164 (3.0)
Deficiency Anemias	16,710 (3.2)	11,670 (3.2)	1,672 (3.2)	3,368 (3.2)
Fluid and Electrolytes-				
Abnormality	23,787 (4.5)	16,682 (4.5)	2,364 (4.5)	4,741 (4.5)
Myocardial Infarction	22,396 (4.3)	15,669 (4.3)	2,247 (4.3)	4,480 (4.2)
Cerebrovascular Disease	12,492 (2.4)	8,802 (2.4)	1,150 (2.2)	2,540 (2.4)
Dementia	6,599 (1.3)	4,649 (1.3)	654 (1.3)	1,296 (1.2)
Connective Tissue Disease-				
Rheumatic Disease	4,600 (0.9)	3,245 (0.9)	457 (0.9)	898 (0.8)
Peptic Ulcer Disease	6,274 (1.2)	4,377 (1.2)	653 (1.3)	1,244 (1.2)
Paraplegia and Hemiplegia	3,970 (0.8)	2,811 (0.8)	351 (0.7)	808 (0.8)
Cancer	42,076 (8.0)	29,557 (8.0)	4,125 (8.0)	8,394 (7.9)
Metastatic Carcinoma	14,982 (2.8)	10,488 (2.8)	1,541 (3)	2,953 (2.8)
AIDS/HIV	952 (0.2)	670 (0.2)	87 (0.2)	195 (0.2)

Note. AIDS/HIV = Acquired Immune Deficiency Syndrome/Human Immunodeficiency Virus. Values are *n* (%) for categorical variables and mean (SD) for continuous variables. Percentages may not total 100 due to rounding.

Table S5. List of tuned hyperparameters for each machine learning model.

Model	Hyperparameters
Logistic Regression	*C*, *fit_intercept*, *solver*, *max_iter*, *tol*
Decision Tree	*max_depth*, *min_samples_split*, *min_samples_leaf*, *ccp_alpha*, *criterion*, *max_features*
Random Forest	*max_samples*, *n_estimators*, *max_depth*, *min_samples_split*, *min_samples_leaf*, *ccp_alpha*, *criterion*, *max_features*, *class_weight*
XGBoost	*n_estimators*, *max_depth*, *learning_rate*, *subsample*, *colsample_bytree*, *min_child_weight*, *gamma*, *reg_alpha*, *reg_lambda*, *scale_pos_weight*, *tree_method*
LGBM	*n_estimators*, *learning_rate*, *min_split_gain*, *max_depth*, *num_leaves*, *min_child_samples*, *min_child_weight*, *subsample*, *colsample_bytree*, *reg_alpha*, *reg_lambda*, *boosting_type*
CatBoost	*iterations*, *learning_rate*, *depth*, *l2_leaf_reg*, *border_count*, *bagging_temperature*, *random_strength*, *subsample*, *leaf_estimation_iterations*
Artificial Neural Network	*activation*, *alpha*, *learning_rate_init*, *man_iter*, *beta_1*, *beta_2*, *epsilon*

Table S6. Extended performance metrics for machine learning model comparison.

**Metrics**	LR	DT	RF	XGBoost	LGBM	CatBoost	MLP-ST (100,50)	MLP-ST (100,50,25)
AUC-ROC [95% CI]	0.8385 [0.8355, 0.8416]	0.8264 [0.8233, 0.8295]	0.8509 [0.8480, 0.8537]	**0.8608** [0.8580, 0.8635]	0.8569 [0.8541, 0.8597]	0.8594 [0.8566, 0.8622]	0.8361 [0.8331, 0.8390]	0.8423 [0.8393, 0.8452]
AUC-PR	0.5274	0.5021	0.5610	**0.5863**	0.5768	0.5795	0.5131	0.5297
Accuracy	0.7659	0.7330	0.8226	0.8259	0.7466	0.7725	0.8191	**0.8404**
Balanced Accuracy	0.7605	0.7484	0.7496	0.7640	0.7765	**0.7803**	0.7248	0.7077
Sensitivity (Recall)	0.7522	0.7720	0.6378	0.6691	**0.8224**	0.7923	0.5805	0.5045
Specificity	0.7688	0.7248	0.8614	0.8589	0.7306	0.7684	0.8692	**0.9110**
PPV (Precision)	0.4060	0.3709	0.4917	0.4991	0.3908	0.4182	0.4825	**0.5435**
NPV	0.9366	0.9380	0.9188	0.9251	**0.9514**	0.9463	0.9079	0.8974
F_1-score	0.5274	0.5010	0.5553	**0.5717**	0.5298	0.5475	0.5270	0.5233
$F_{0.5}$-score	0.4472	0.4139	0.5153	0.5258	0.4366	0.4618	0.4994	**0.5352**
F_2-score	0.6426	0.6347	0.6020	0.6264	**0.6736**	0.6721	0.5578	0.5118
Log Loss	0.5047	0.5121	0.3971	0.3792	0.5040	0.4690	0.3857	**0.3593**
MCC	0.4225	0.3917	0.4527	**0.4732**	0.4350	0.4521	0.4190	0.4280
Brier Score	0.1640	0.1729	0.1257	0.1208	0.1684	0.1544	0.1240	**0.1133**
Cohen's Kappa	0.3898	0.3481	0.4468	**0.4653**	0.3851	0.4143	0.4163	0.4276

Note. AUC-ROC = Area Under the Receiver Operating Characteristic Curve; AUC-PR = Area Under the Precision-Recall Curve; PPV = Positive Predictive Value; NPV = Negative Predictive Value; MCC = Matthews Correlation Coefficient; MLP = Multi-Layer Perceptron; ST = SMOTETomek.

Table S7. Mapping of triage chief complaints to medical specialty-based categories.

Group	Triage Chief Complaints
Cardiovascular	Douleur thoracique angineuse, Douleur thoracique non angineuse, Palpitation - pouls irrégulier, Hypertension, Syncope/Pré-syncope, Arrêt cardiaque (non traumatique), Arrêt cardiaque (traumatique), Oedème bilatéral des jambes, Membre sans pouls et froid
Respiratory	Dyspnée, Toux/Congestion, Toux et fièvre, Hémoptysie, Hyperventilation, Inhalation nocive, Corps étranger dans les voies respiratoire, Arrêt respiratoire
Gastrointestinal	Douleur abdominale, Nausées/vomissements, Diarrhée, Rectorragie/Méléna, Constipation, Douleur au flanc, Hématémèse, Masse/Ballonnement abdominal, Douleur rectale/périnéale, Corps étranger oral/oesophagien, Corps étranger rectal, Ictère/Jaunisse
Neurological	Céphalée, Vertige, Faiblesse d'un membre/Sympt AVC, Convulsion, Confusion, Altération du niveau de conscience, Trouble d'équilibre/Ataxie, Tremblements, Hypoesthésie/Paresthésie
Musculoskeletal	Douleur au membre inférieur, Douleur dorsale, Oedème/Douleur au cou, Douleur au membre supérieur, Blessure au membre inférieur, Blessure au membre supérieur, Gonflement articulaire, Trauma dos/rachis, Douleur faciale (non-traumatique/non dentaire)
Trauma	Traumatisme crânien, Traumatisme majeur - non pénétrant, Traumatisme majeur - pénétrant, Traumatisme facial, Traumatisme thoracique - non pénétrant, Traumatisme thoracique - pénétrant, Traumatisme abdominal - non pénétrant, Traumatisme abdominal - pénétrant, Traumatisme au cou, Traumatisme oculaire, Traumatisme de l'oreille, Traumatisme nasal, Traumatisme génital, Traumatisme anal/rectal, Amputation, Lacération/Plaie punctiforme, Brûlure, Abrasion, Électrisation, Morsure

Continued on next page

Continued from previous page

Group	Triage Chief Complaints
Genitourinary	Symptômes d'infection urinaire, Rétention urinaire, Hématurie, Douleur ou oedème au scrotum, Douleur ou prurit vaginal (e), Saignement vaginal, Écoulement vaginal, Écoulement/Lésion génitale, Oedème au pénis, Oedème vulvaire, Corps étranger vaginal, Problème de grossesse $<$ 20 sem., Problème de grossesse $>$ 20 sem., Problèmes menstruels, Rougeur/Oedème du sein, Polyurie, Agression sexuelle, Douleur/Masse à l'aine, Oligo-anurie
ENT/Ophthalmological	Problème de vision, Douleur à la gorge, Douleur oculaire, Difficulté à avaler/Dysphagie, Otalgie, Épistaxis, Écoulement/rougeur oculaire, Photophobie, Corps étranger oculaire, Perte d'audition, Oedème périorbitaire, Écoulement de l'oreille, Acouphène, Réévaluation oculaire, Exposition de l'oeil à un produit chimique, Corps étranger dans l'oreille, Corps étranger nasal, Problèmes dentaires ou de gencives, Diplopie, Congestion nasale/Rhinite
Dermatological	Oedème ou rougeur localisé(e), Éruption cutanée, Nodules, bosses, ampoules, Autre condition de la peau, Membre chaud et rouge, Prurit, Corps étranger (peau), Éliminer infestation, Engelures
Psychiatric	Anxiété/Crise situationnelle, Idées suicidaires, Comportement bizarre, Dépression ou Automutilation, Tentative de suicide, Hallucinations, Comportement violent/homicidaire, Insomnie, Problème social, Inquiétude face à la sécurité du patient
Infectious	Fièvre, Symptômes d'IVRS, Diarrhée et fièvre, COVID-19 suspecté/Exposition à une maladie contagieuse
Metabolic/Endocrine	Hyperglycémie, Hypoglycémie, Résultats de laboratoire anormaux, Anorexie
Toxicological	Usage de toxiques/drogues, Intoxication, Symptômes de sevrage, Exposition au sang ou autre liquide biologique, Exposition à un produit chimique

Continued on next page

Continued from previous page

**Group**	**Triage Chief Complaints**
General/Systemic	Plaintes mineures, Cyanose, Hypothermie, Réaction allergique, Faiblesse générale, Oedème généralisé, Ecchymose spontanée, Teint pâle/Anémie, Quasi-noyade, Piqûre d'insecte, Hoquet
Procedural/Follow-up	Référence pour consultation, Réévaluation d'une plaie, Test d'imagerie diagnostique, Problème d'appareil médical, Complications postopératoires, Demande d'ordonnance ou de médicament, Vérification de plâtre, Changement de pansement, Enlever points/agrafes, Enlever bague

Table S8. List of unique values for features with many distinct values.

**Feature**	**Values**
Arrival Mode	Ambulant, Ambulance, Autre, Police, Fourgon (prison), Hélicoptère
Patient Origin	Domicile, Hôpital, Autre, Clinique, Sans domicile, Bureau, CHSLD (Centre d'hébergement de soins de longue durée), Résidence Grand-Nord, Centre de réadaptation, Hôpital Royal-Victoria, CLSC (Centre local de services communautaires), Centre de détention, Hôpital Général de Montreal

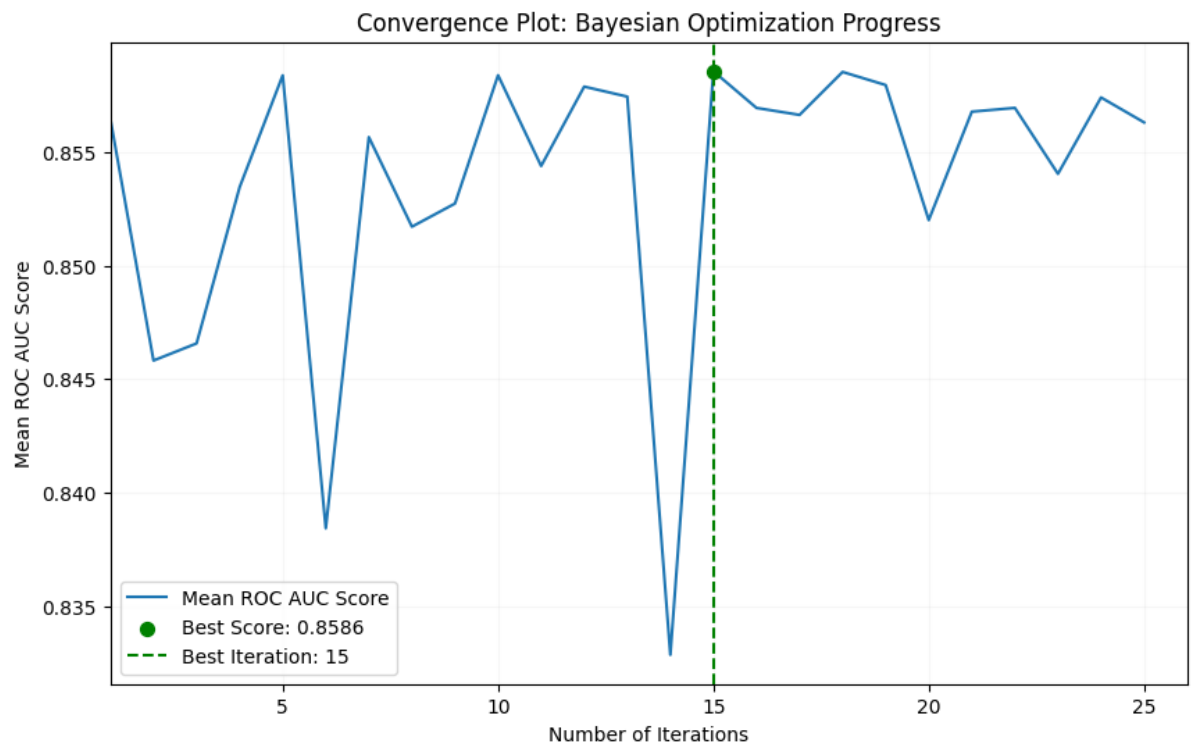

(a) XGBoost

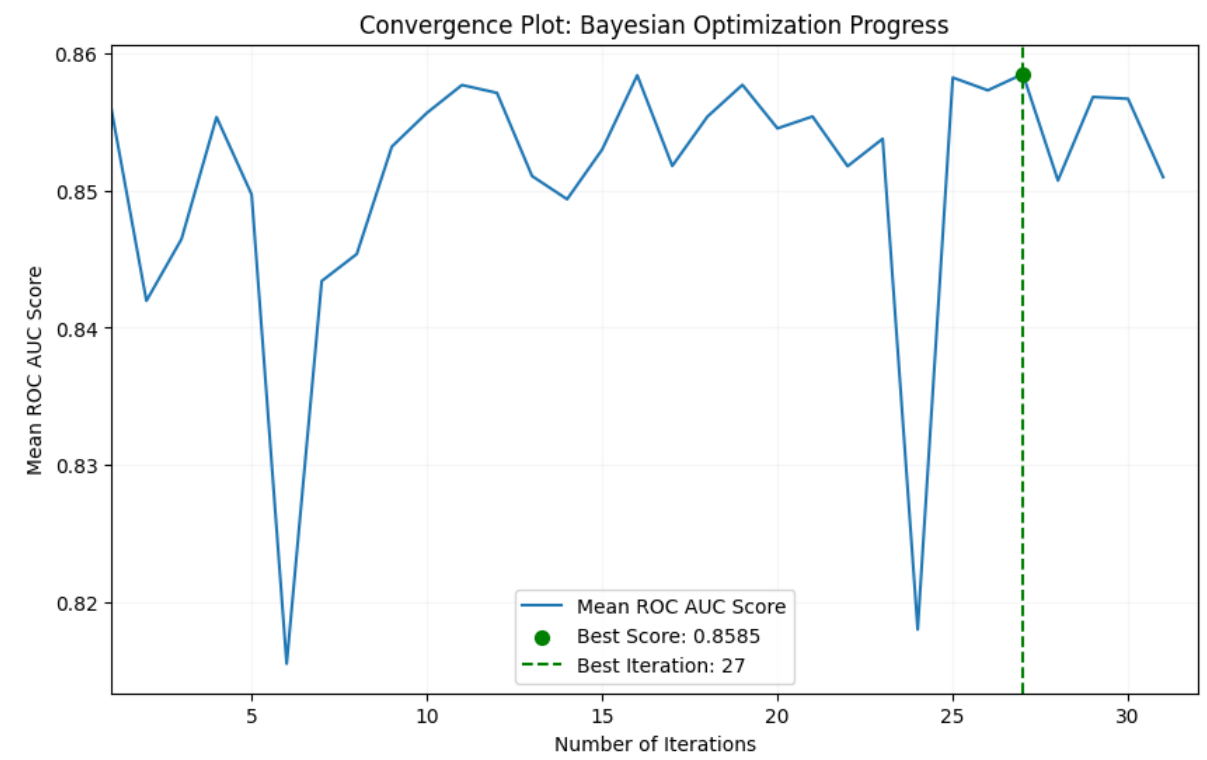

(b) LGBM

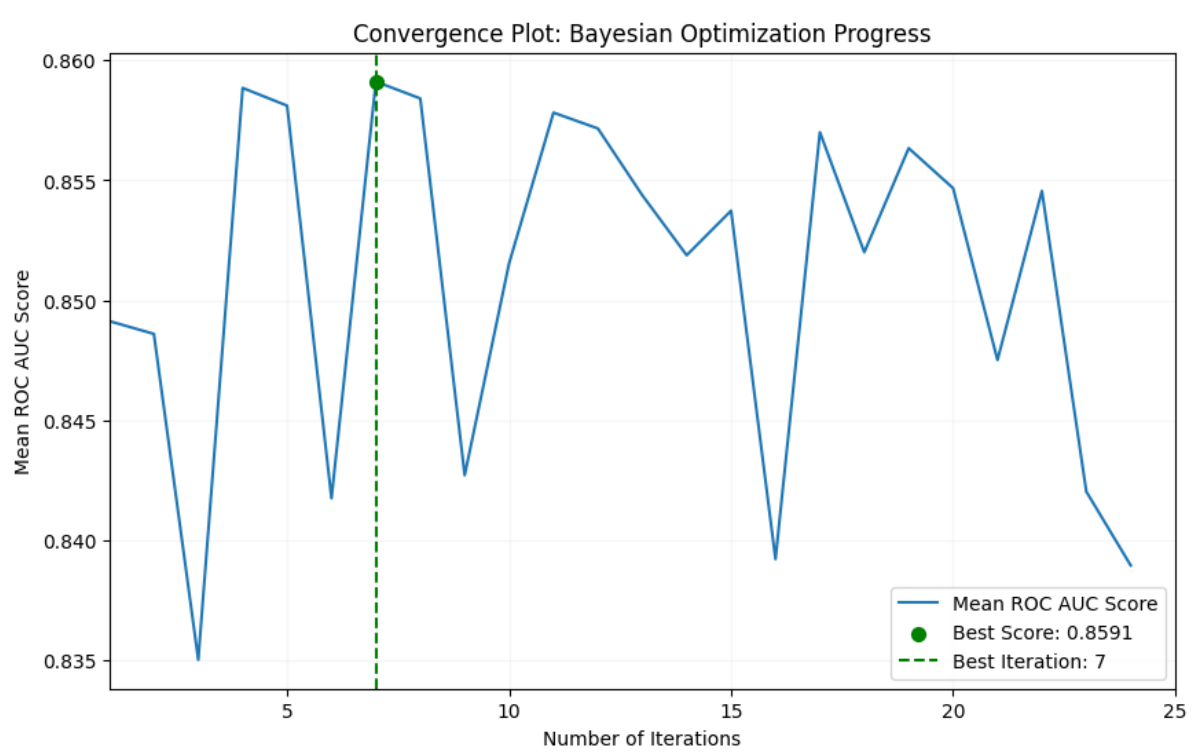

(c) CatBoost

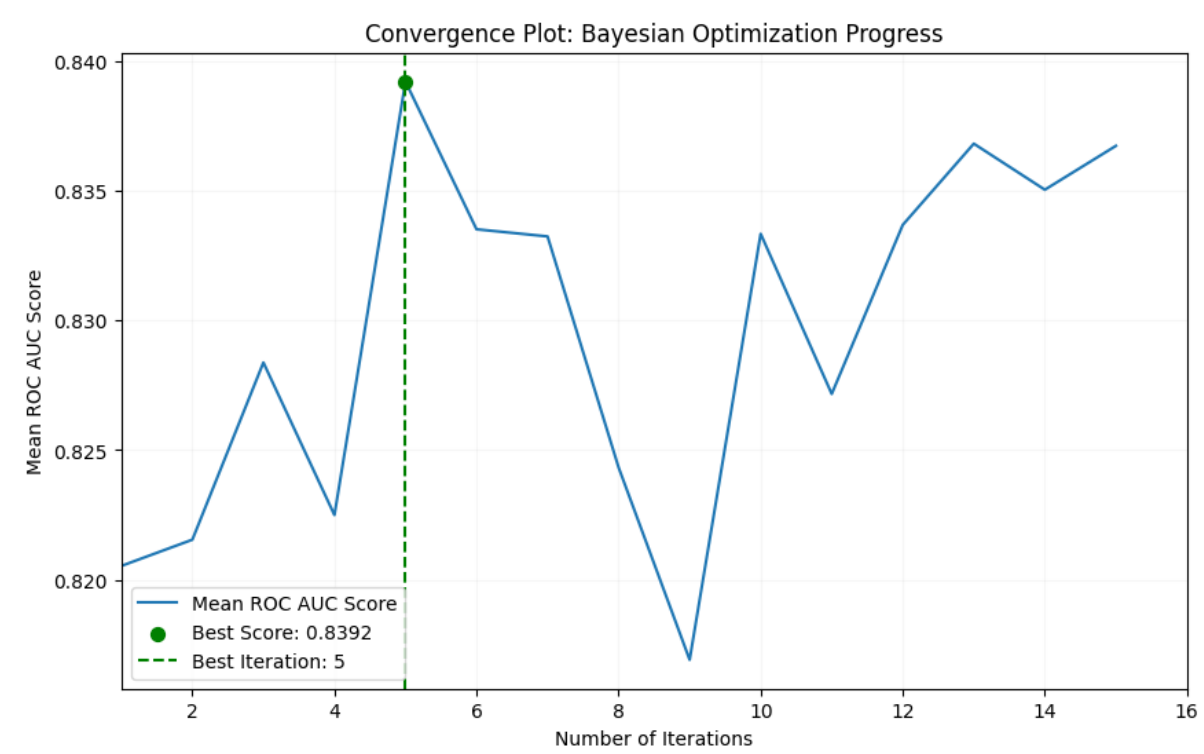

(d) MLP-ST (100,50,25)

Figure S1. Convergence plots illustrating the hyperparameter tuning progress for the top-performing machine learning models. Each graph tracks the cross-validated performance metric, mean area under the receiver operating characteristic curve (ROC AUC), across successive evaluation iterations. The markers indicate the specific iteration where the optimal hyperparameter configuration was identified to finalize each model.

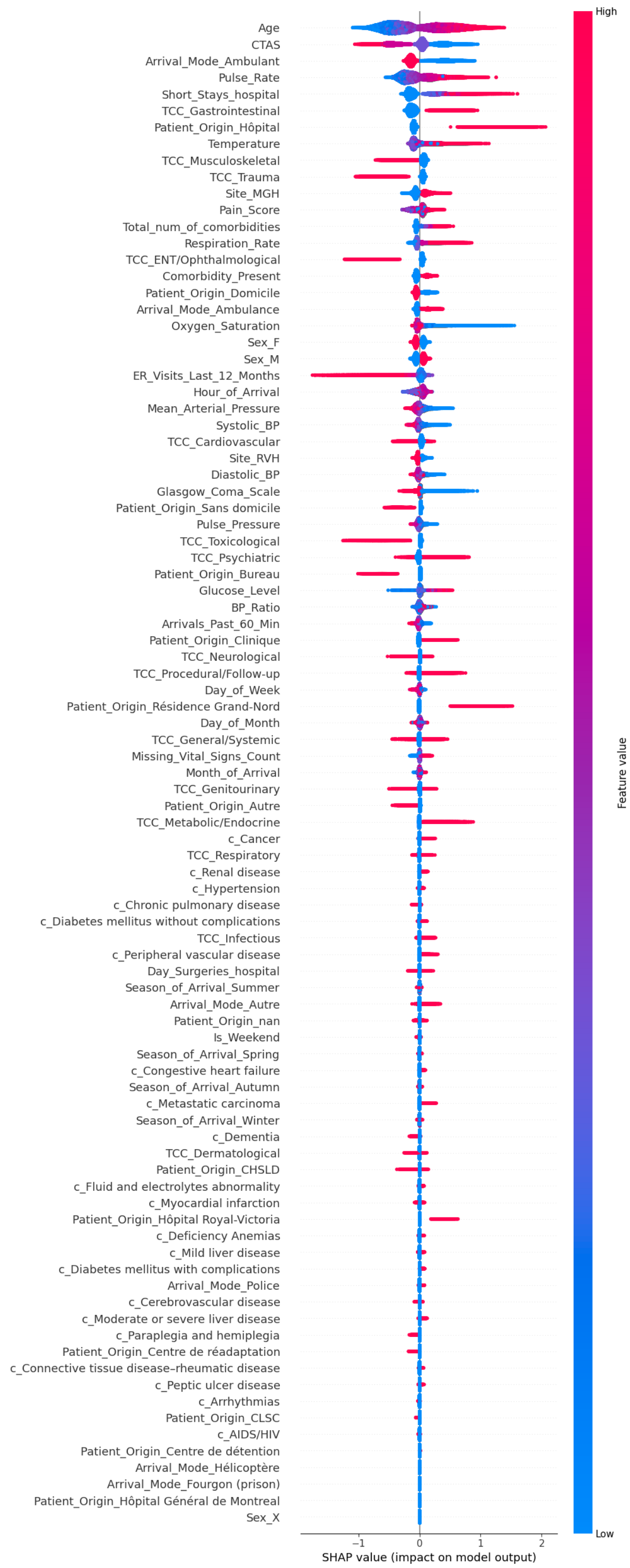

Figure S2. SHAP summary beeswarm plot illustrating feature importance and impact direction for all model features.

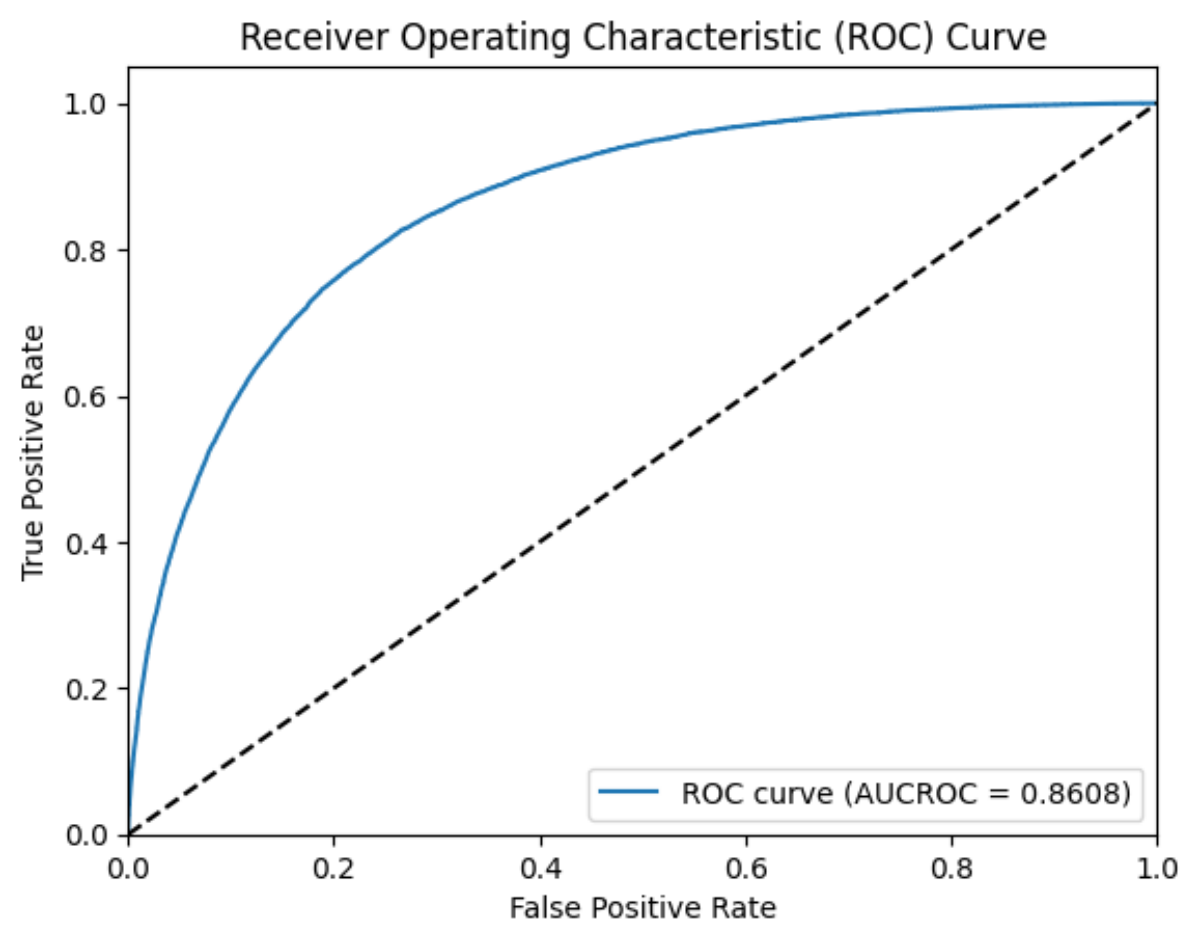

(a) XGBoost

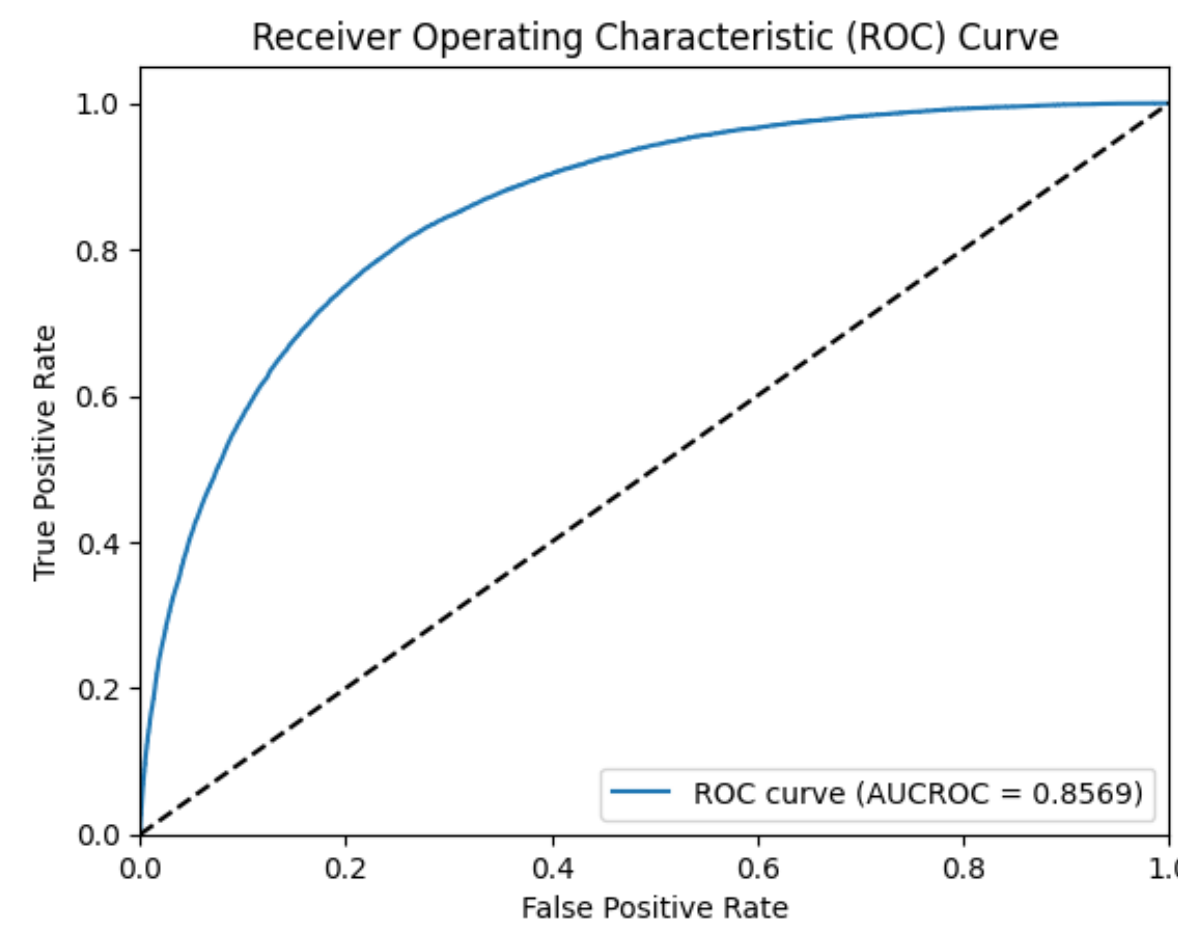

(b) LGBM

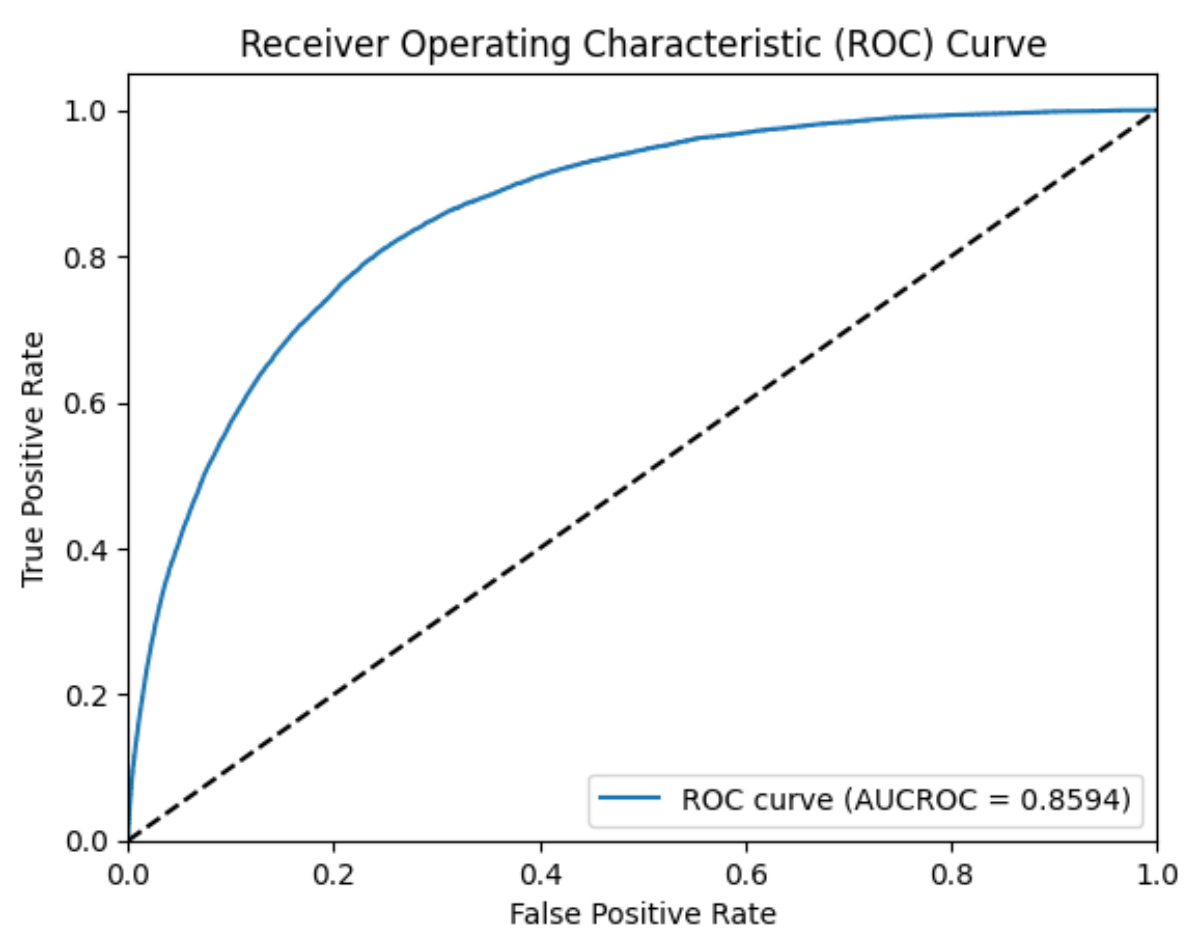

(c) CatBoost

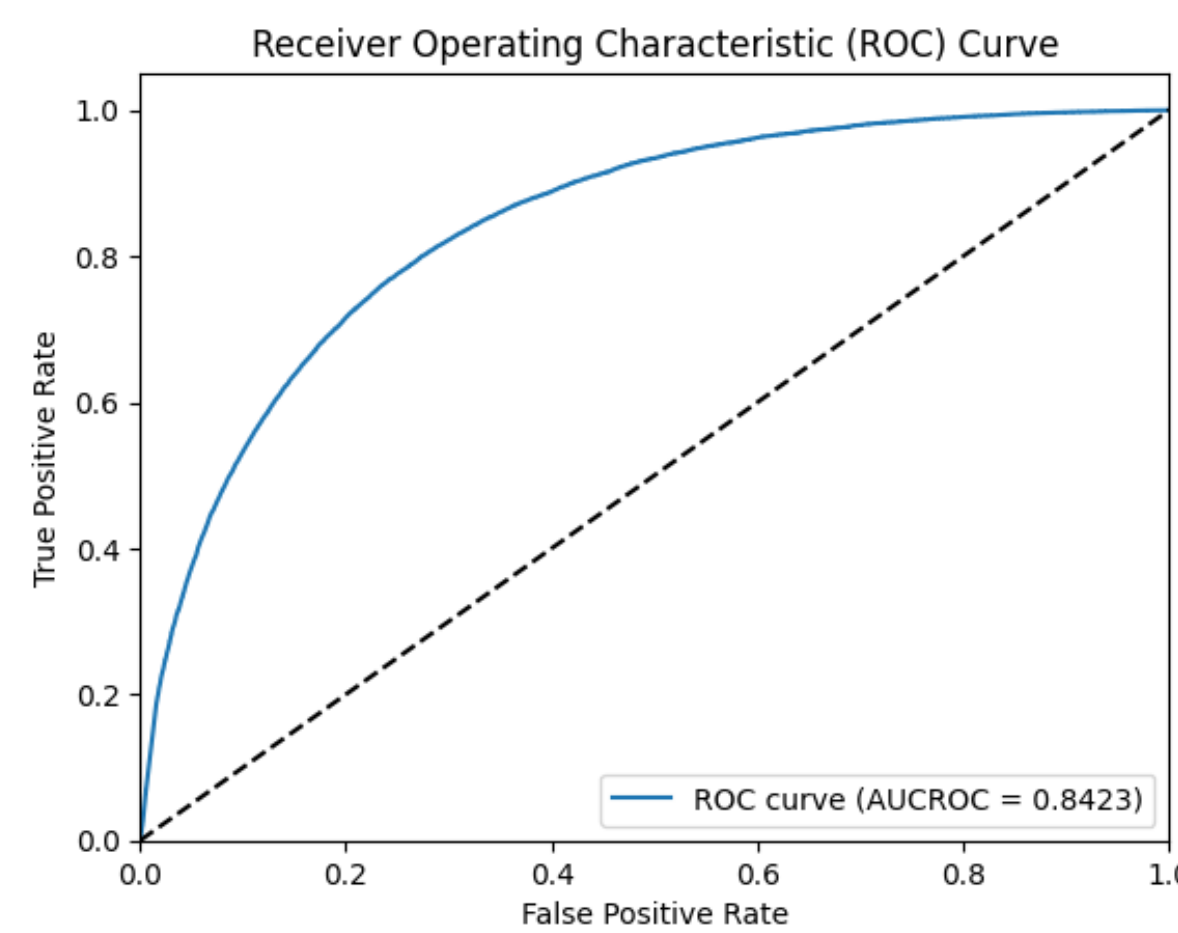

(d) MLP-ST (100,50,25)

Figure S3. Receiver Operating Characteristic (ROC) curves for the top-performing machine learning models. Evaluated on the test set.

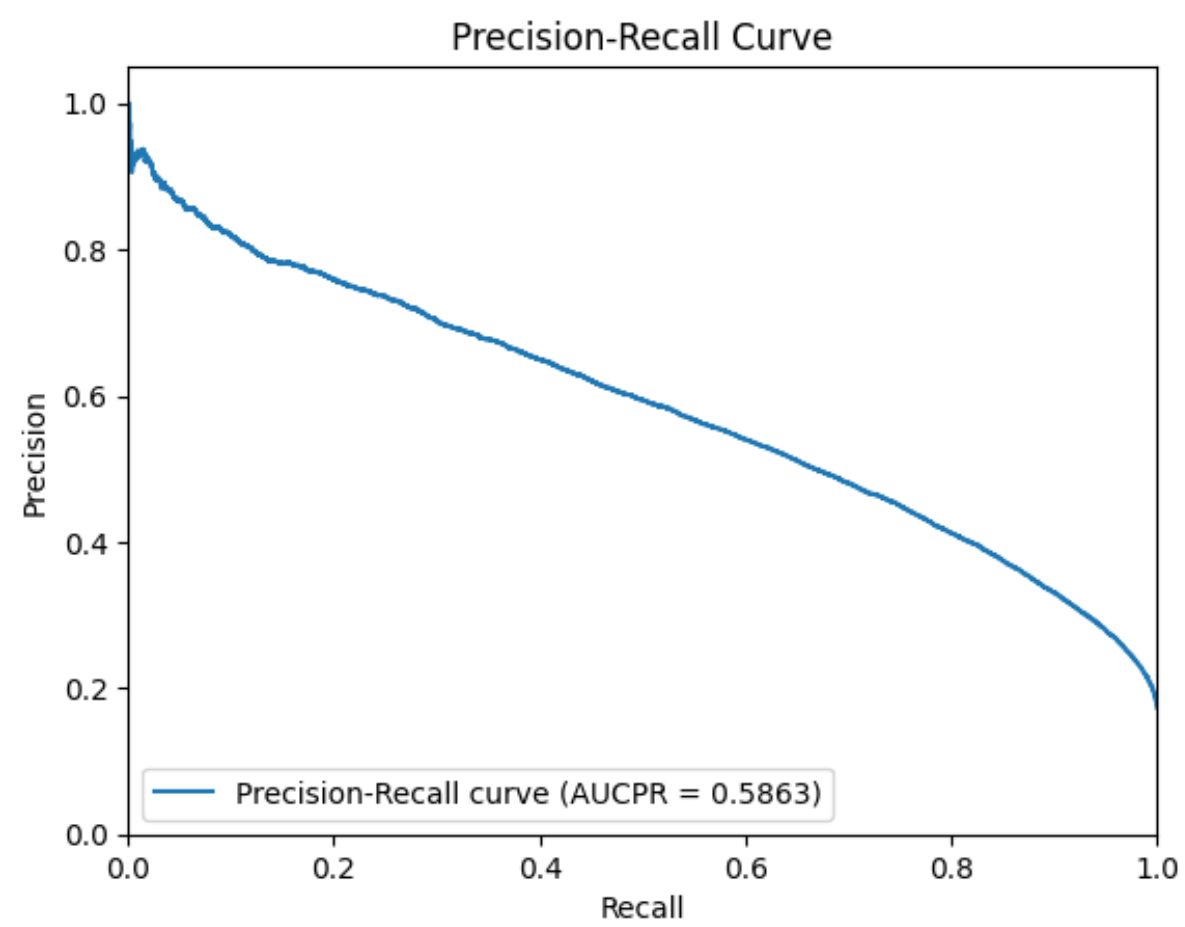

(a) XGBoost

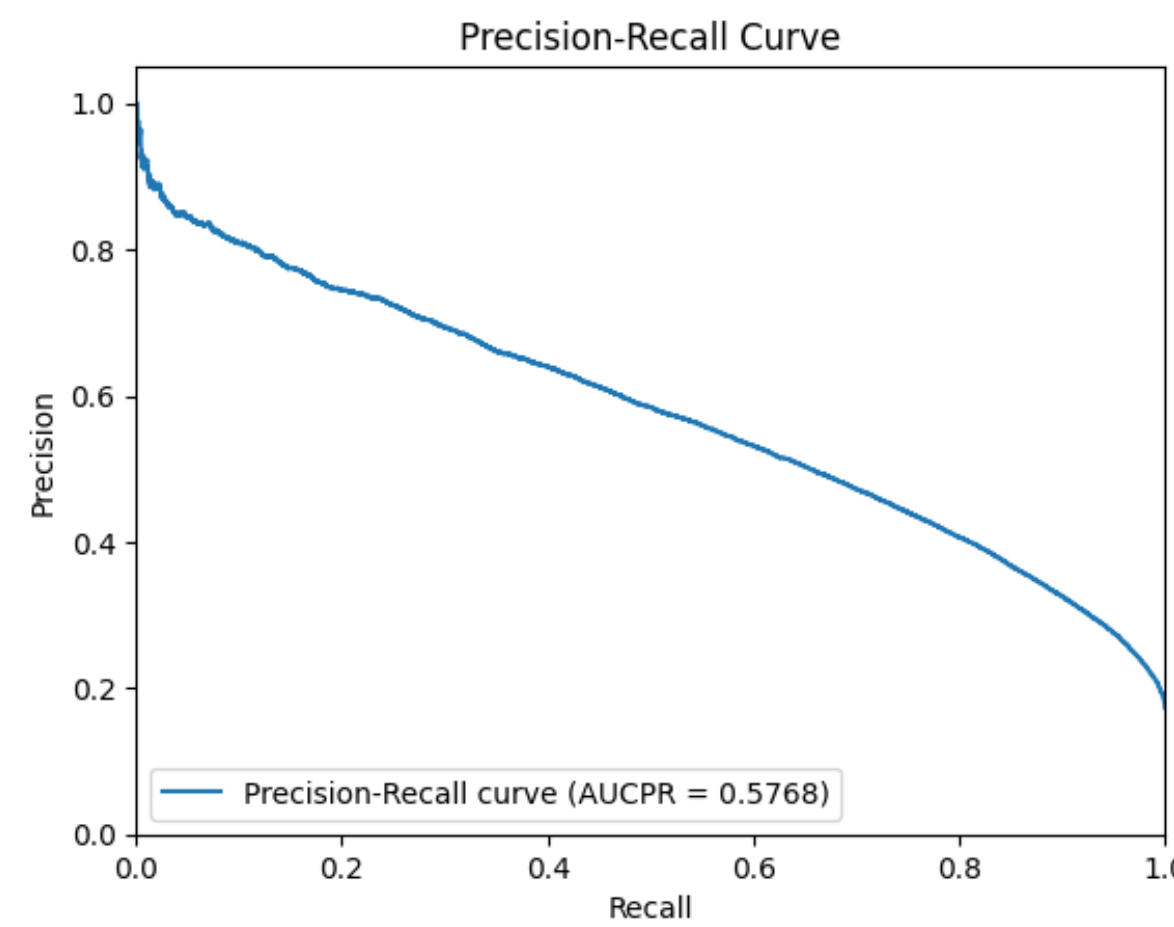

(b) LGBM

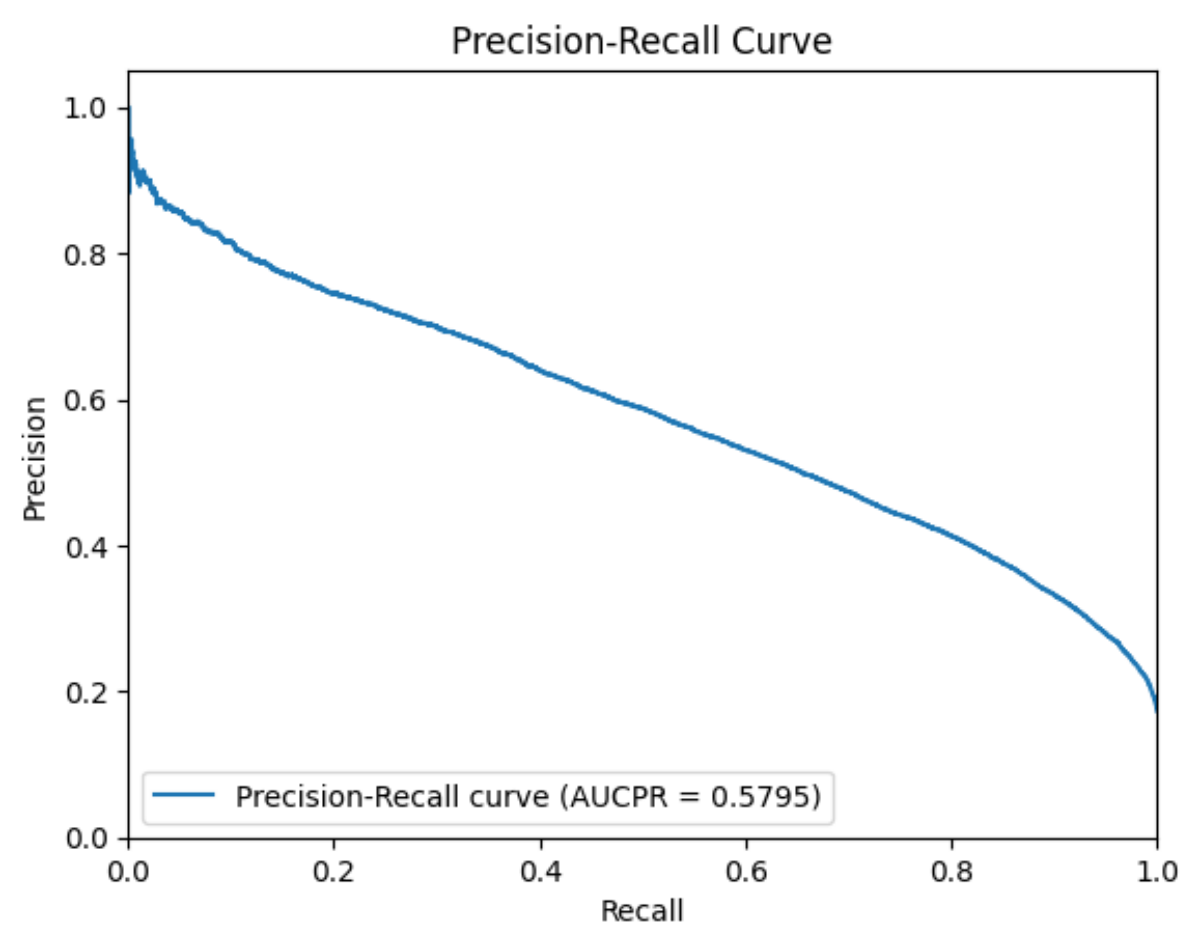

(c) CatBoost

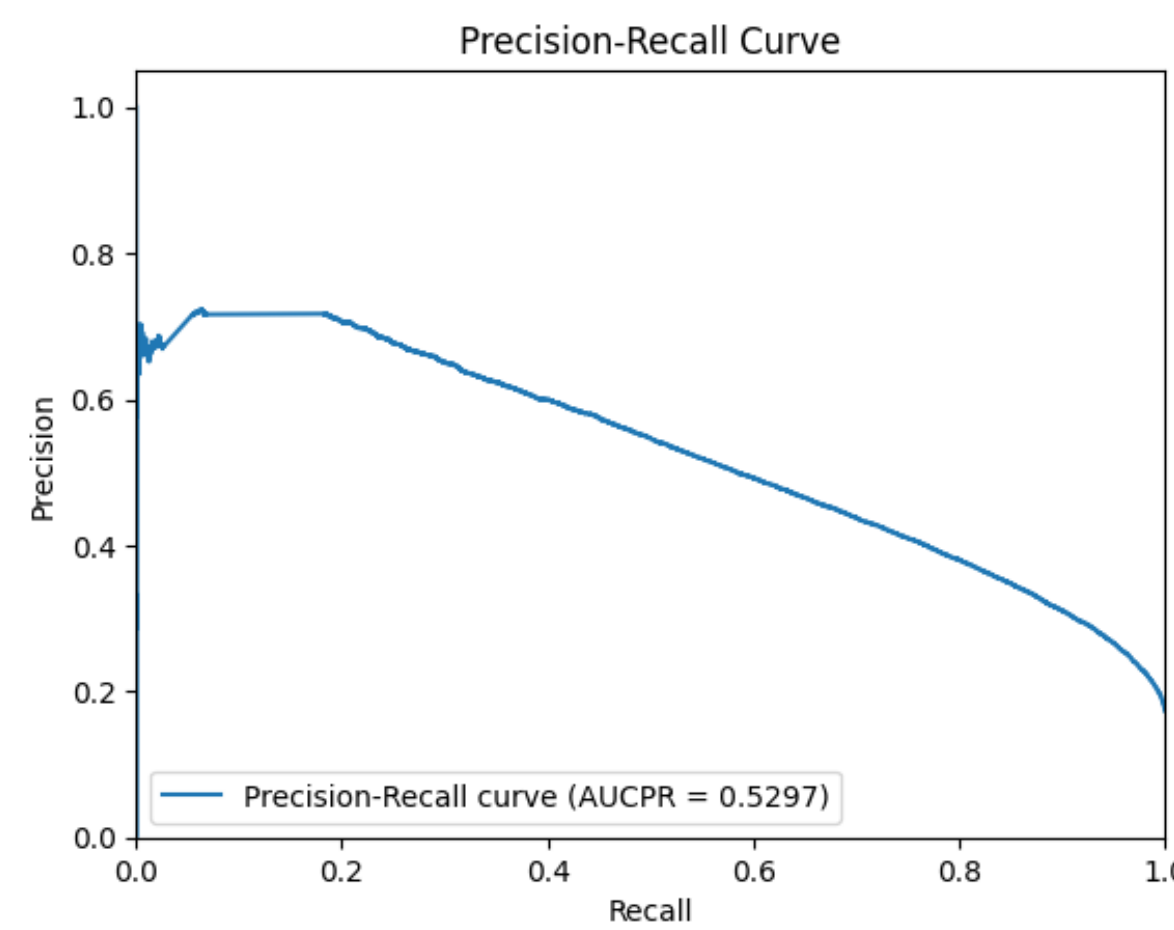

(d) MLP-ST (100,50,25)

Figure S4. Precision-Recall (PR) curves for the top-performing machine learning models. Evaluated on the test set.

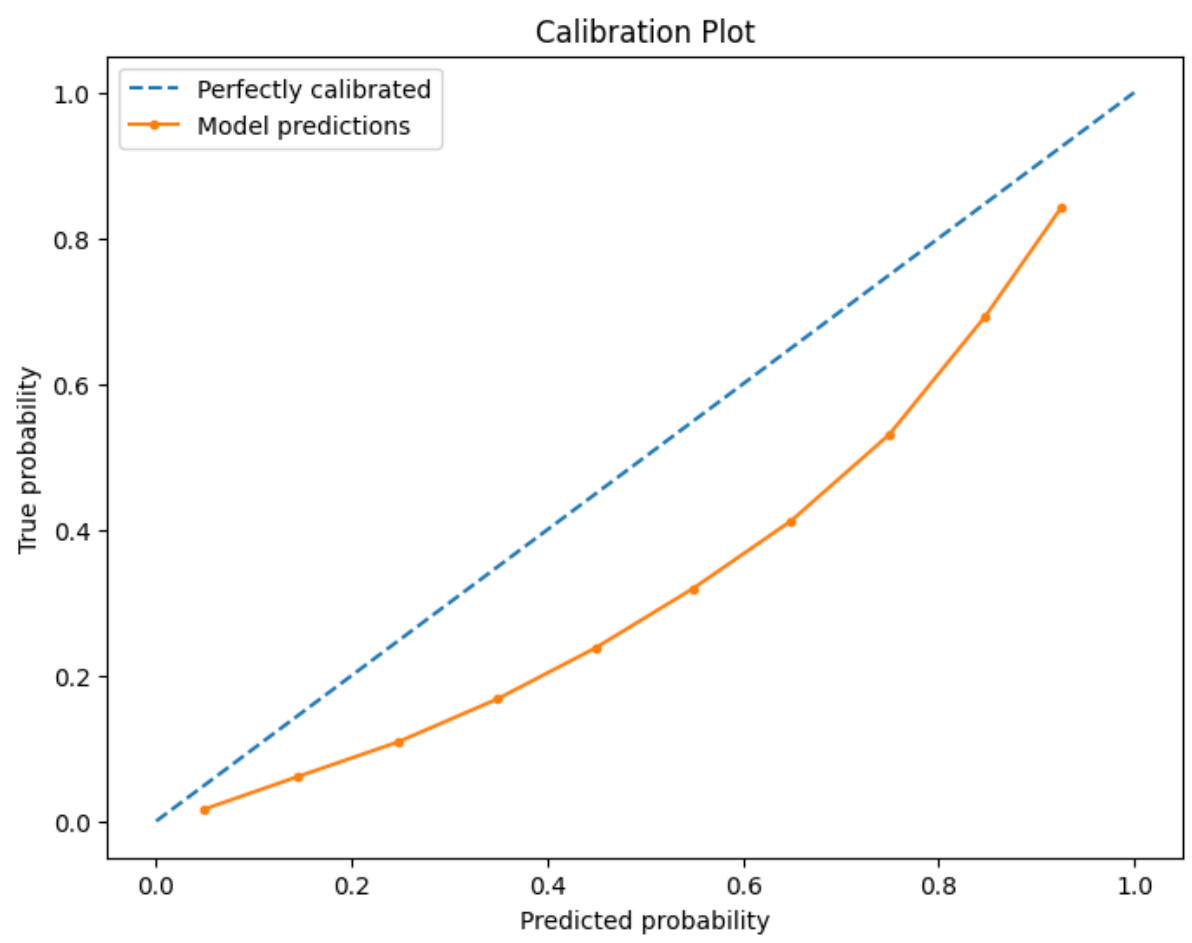

(a) XGBoost

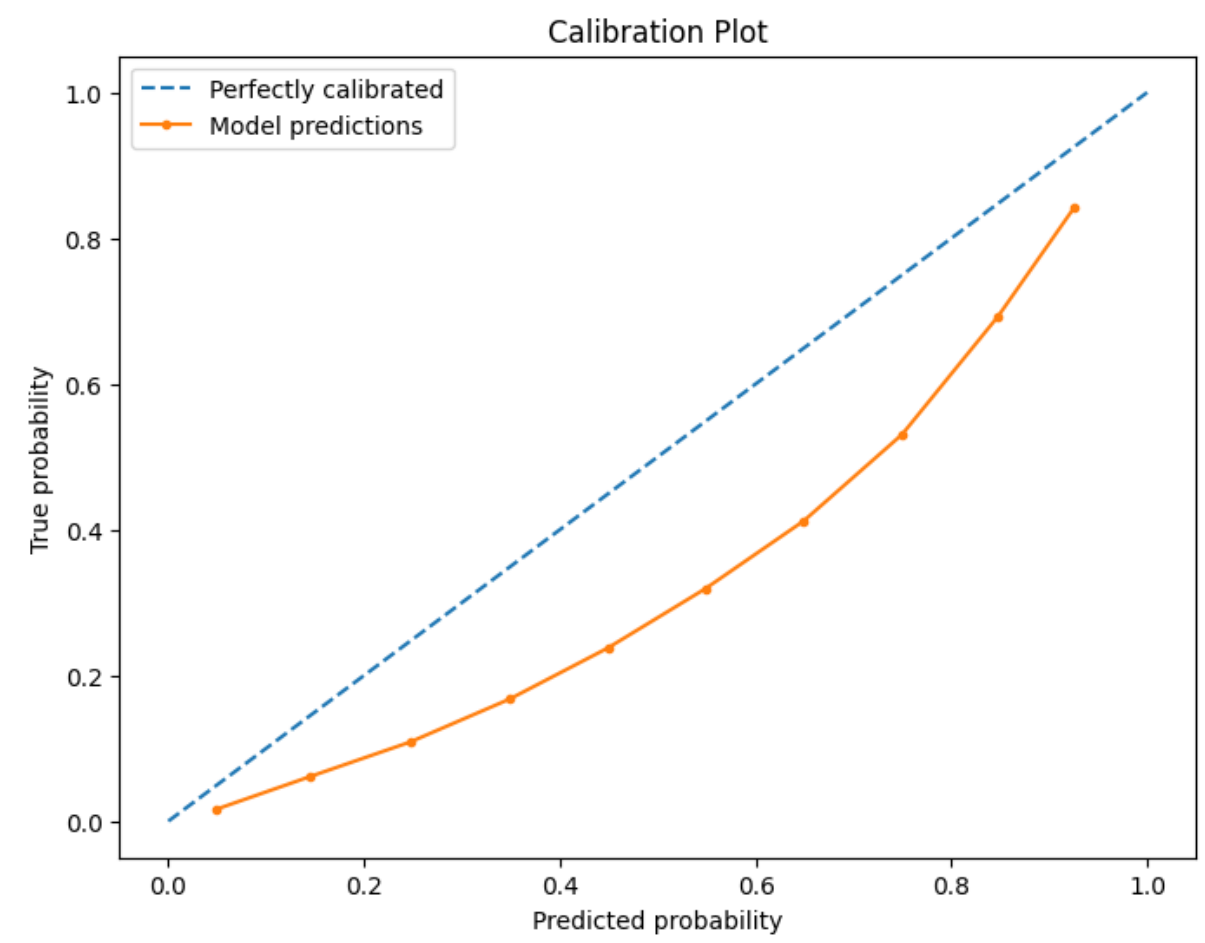

(b) LGBM

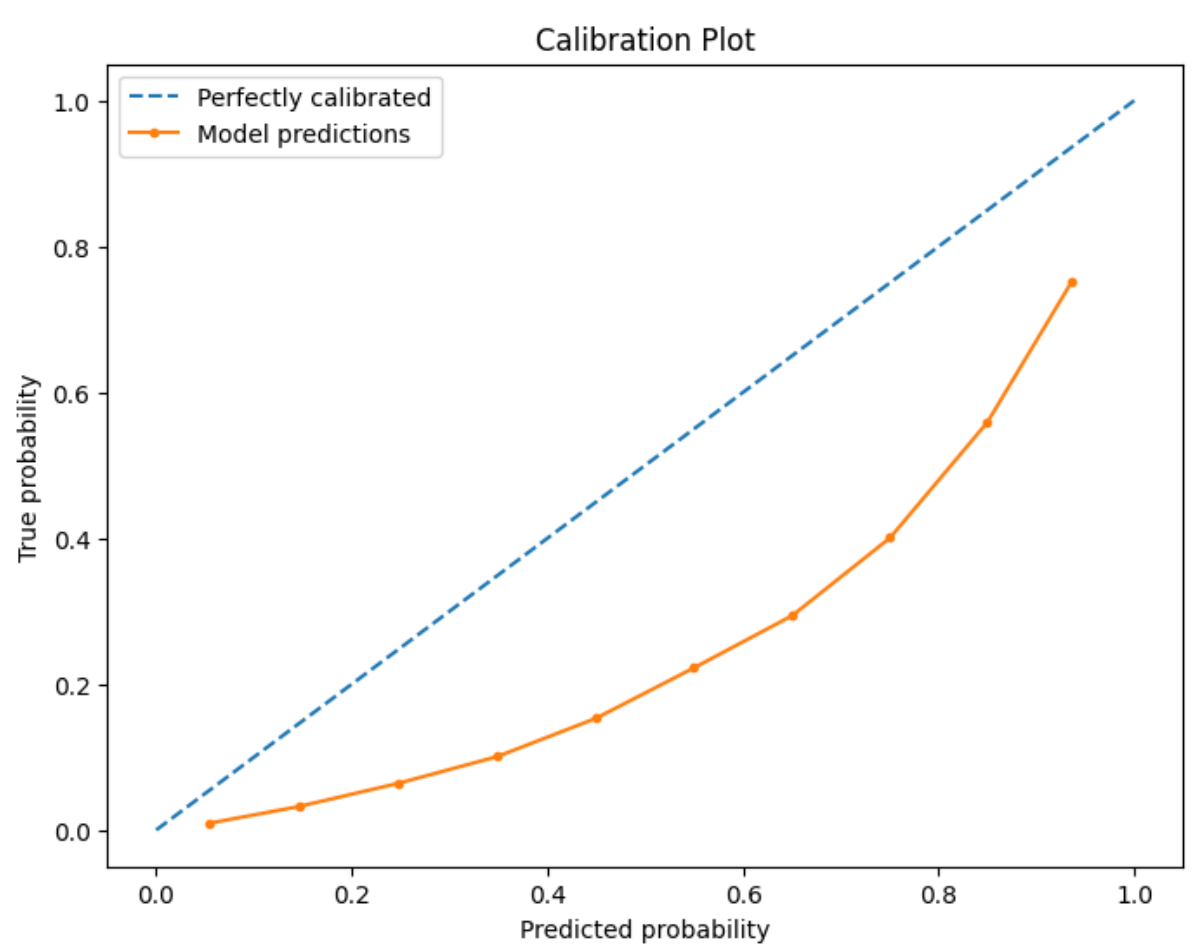

(c) CatBoost

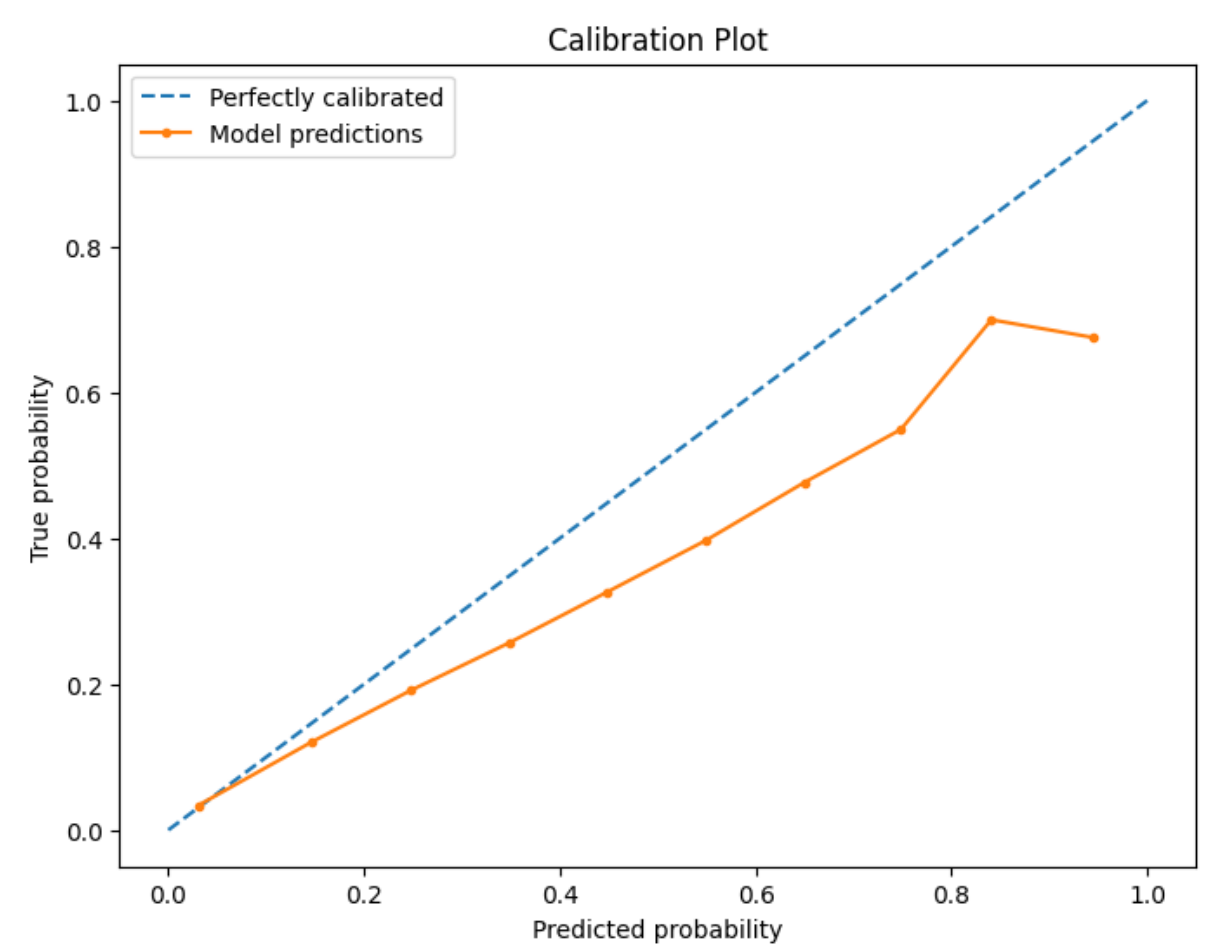

(d) MLP-ST (100,50,25)

Figure S5. Calibration curves for the top-performing machine learning models. Evaluated on the test set.
